# The AAA+ ATPase p97, a cellular multitool

**DOI:** 10.1042/BCJ20160783

**Published:** 2017-08-17

**Authors:** Lasse Stach, Paul S. Freemont

**Affiliations:** Section of Structural Biology, Department of Medicine, Imperial College London, London, U.K.

## Abstract

The AAA+ (ATPases associated with diverse cellular activities) ATPase p97 is essential to a wide range of cellular functions, including endoplasmic reticulum-associated degradation, membrane fusion, NF-κB (nuclear factor kappa-light-chain-enhancer of activated B cells) activation and chromatin-associated processes, which are regulated by ubiquitination. p97 acts downstream from ubiquitin signaling events and utilizes the energy from ATP hydrolysis to extract its substrate proteins from cellular structures or multiprotein complexes. A multitude of p97 cofactors have evolved which are essential to p97 function. Ubiquitin-interacting domains and p97-binding domains combine to form bi-functional cofactors, whose complexes with p97 enable the enzyme to interact with a wide range of ubiquitinated substrates. A set of mutations in p97 have been shown to cause the multisystem proteinopathy inclusion body myopathy associated with Paget's disease of bone and frontotemporal dementia. In addition, p97 inhibition has been identified as a promising approach to provoke proteotoxic stress in tumors. In this review, we will describe the cellular processes governed by p97, how the cofactors interact with both p97 and its ubiquitinated substrates, p97 enzymology and the current status in developing p97 inhibitors for cancer therapy.

## Introduction

**Figure 1.**
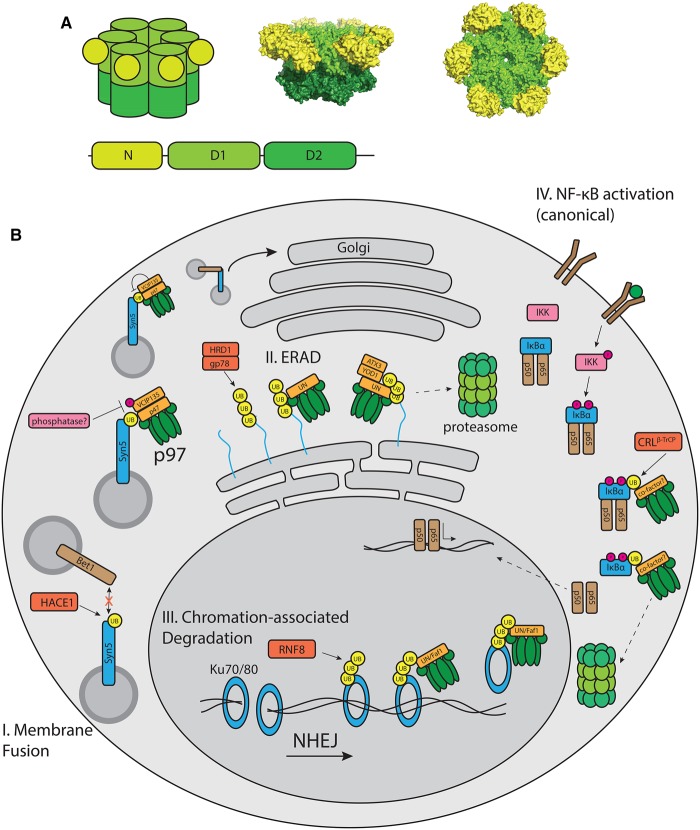
Cellular processes governed by p97. (**A**) Top: schematic of the p97 architecture, side and top views of the cryo-EM structure of ADP-bound p97 (PDB: 5FTK). Bottom: domain organization of p97, color-coded as above. (**B**) Well-characterized cellular process requiring p97 activity. (I) Post-mitotic membrane fusion via the p97/p47 pathway. (II) Endoplasmic reticulum-associated degradation. (III) Removal of KU70/80 from chromatin. (IV) NF-κB activation via the canonical pathway. Proteins are color-coded according to their function. p97 and the proteasome are in green, ubiquitinated p97 substrates are blue, cognate E3 ubiquitin ligases decorating p97 substrates are red, phosphorylation signaling proteins are in pink, ubiquitin is in yellow and other proteins are in brown.

The human AAA+ (ATPases associated with diverse cellular activities) ATPase p97, also known as valosin-containing protein (VCP) and homologs Cdc48 (cell division cycle protein 48) in *Saccharomyces cerevisiae*, Ter94 (transitional endoplasmic reticulum ATPase) in *Drosophila melanogaster* and VAT (VCP-like ATPase) in *Thermoplasma acidophilum*, is highly conserved across all domains of life [1]. It contains an amino-terminal N-domain, two AAA ATPase domains and a C-terminal unstructured extension. Six p97 protomers form a homohexamer with two planar ATPase rings stacked on top of each other (Figure 1A) [2,3]. The enzyme performs many important cellular functions, highlighted by its conservation across diverse species and its abundance [4]. In *Xenopus leavis*, the protein makes up ∼1% of cytosolic protein [5,6].

The cellular processes in which p97 is implicated include protein degradation via the ubiquitin proteasome system (UPS), DNA repair, DNA replication, cell cycle regulation, NF-κB (nuclear factor kappa-light-chain-enhancer of activated B cells) activation, endoplasmic reticulum-associated degradation (ERAD), mitochondria-associated degradation, Golgi formation and autophagy, reviewed in ref. [7]. In all of these processes, ubiquitination of p97 substrates is a common theme. The enzyme converts the energy of ATP hydrolysis into mechanical energy to extract ubiquitinated proteins from lipid membranes or macromolecular complexes. While p97 itself has a weak affinity for ubiquitin, it interacts with its targets primarily via cofactors. It is the variety of these cofactors that allow a single machine such as an ATPase to play a role in the diverse processes mentioned above, reviewed in ref. [8,9]. The large proportion of p97 cofactors identified so far are part of the ubiquitin system, either ubiquitin ligases or DUBs (deubiquitinases) or ubiquitin-binding proteins that function as adapters [7]. Mutations in p97 have been associated with familial amyotrophic lateral sclerosis and inclusion body myopathy associated with Paget's disease of bone and frontotemporal dementia (IBMPFD), a multisystem disease that is associated with abnormally active p97 on a molecular level leading to neurodegenerative defects and muscular weakness at the physiological level [10,11]. This review will provide an overview of the cellular processes p97 is involved in, what role its cofactors play in such processes, the molecular mechanism and regulation of the enzyme, as well as efforts to target p97 in cancer chemotherapy. Where possible, connections will be made between these different aspects to provide a more integrated picture.

## Cellular processes associated with p97

The p97 AAA ATPase has been found to be involved in a large variety of cellular processes. In this section, we will describe some of the main pathways and cellular functions that p97 has been directly implicated in.

### Endoplasmic reticulum-associated degradation

The ERAD pathway serves as a quality control system for nascent proteins entering the secretory pathway, a pathway in which the UPS and p97 play central roles (Figure 1B) [12]. Nascent peptide chains that fail to fold properly in the ER require retrotranslocation into the cytosol where they are degraded by the proteasome. It is in the energetically unfavorable retrotranslocation event in which p97 plays its role in ERAD [13,14].

Ubiquitin binding, ATP hydrolysis and p97 cofactor binding all play a role in retrotranslocation. The heterodimeric UFD1–NPL4 (ubiquitin fusion degradation protein 1 and nuclear protein localization protein 4 homolog) complex possess both ubiquitin-binding domains and p97-interacting motifs [15–18]. It recruits p97 to ubiquitinated substrates at the ER membrane [19]. Another ERAD-associated p97 cofactor is Ubx2 [20]. The protein also recruits p97 to the ER membrane, and specifically, it facilities the interaction between p97 and Hrd1 (Hmg2-regulated degradation), a membrane-anchored ubiquitin ligase which ubiquitinates ERAD substrates and also contains a p97-interacting VBM (VCP-binding motif) [21–24].

The ubiquitin E3 ligase gp78 (also known as the RING-type E3 ubiquitin transferase autocrine motility factor receptor) and the DUBs Ataxin-3 (spinocerebellar ataxia type 3 protein) and YOD1 (also known as ubiquitin thioesterase OTU1 in yeast) are examples of p97 ubiquitin catalytic cofactors. The E3 ligase interacts with p97 through its VIM (VCP-interacting motif) domain and has been shown to act downstream from Hrd1 in the degradation of ERAD substrates [25–27]. The role of the DUB Ataxin-3 is less well defined. This protein contains a poly-glutamine (poly-Q) domain, which causes its aggregation in genetic variants where this sequence is extended [28]. Similar to gp78, Ataxin-3 also interacts with p97 through the arginine/lysine-rich VBM [29,30]. Functionally, the ubiquitin chain-trimming activity of this enzyme has been suggested either to slow down substrate processing [31] or to facilitate the transport of substrates from p97 to the proteasome [32]. The DUB YOD1, which binds to p97 via its UBX (ubiquitin regulatory X) domain, is also associated with ERAD [33]. Its role in ERAD was proposed to be the trimming of polyubiquitin chains to optimize p97 recruitment or the removal of ubiquitin to allow the substrate to be threaded through the p97 pore for unfolding [34].

The complexes at the ER membrane formed by p97 interact primarily with K11- and K48-linked ubiquitin chains. Upon disruption of p97 function or overexpression of a catalytically dead mutant of YOD1, the model ERAD substrate CD3δ (T-cell surface glycoprotein CD3 delta chain), decorated with these types of polyubiquitin, accumulates in the cell [35].

### Chromatin-related functions of p97

The functions of p97 are not limited to extraction of substrates from lipid bilayers such as in ERAD, as p97 has also been shown to be essential in the extraction of proteins from chromatin (reviewed in ref. [36]). Chromatin-related processes where p97 is essential include the disassembly of protein complexes at sites of DNA damage and the unloading of stalled replication helicases. The regulation of DNA double-strand break repair is regulated by extensive post-translational modification events, particularly protein phosphorylation and ubiquitination, which facilitate the assembly of large multiprotein complexes essential to DNA repair [37,38]. Proteins that interact with both types of modification, such as the ubiquitin E3 ligase RNF8 (RING finger protein 8), which contains a pThr-binding FHA (forkhead-associated) domain, facilitate cross-talk between these modifications [39]. Ubiquitination plays a role in complex assembly and disassembly, a process where p97 is required [36]. The removal of proteins from chromatin by p97 is not only limited to the final disassembly of complexes, but also includes the removal of K48-ubiquitinated proteins from complexes to facilitate the binding of downstream factors. Recently discovered examples of protein extraction from chromatin by p97 will be introduced in the following section.

The Ku70/Ku80 (70/80 kDa subunit of Ku antigen) complex, which binds the open ends of DNA double-strand breaks in a first step towards break repair via non-homologous end joining, is a recently discovered p97 substrate (Figure 1B) [40,41]. The Ku70/Ku80 complex forms a ring-like structure with a central cavity for DNA [42]. Following joining of the DNA ends, the protein is trapped around the double helix like a hose clamp and forms a barrier to further processing of the break. Thus, the protein complex is decorated with K48-ubiquitin chains by RNF8, triggering the recruitment of p97 to the site and subsequent removal of Ku70/Ku80 from DNA [41]. There is some redundancy as to which cofactors play a role in this process. Both FAF1 (FAS-associated factor 1) and UFD1–NPL4 have been shown to be sufficient for this process, with UFD1–NPL4 being the more efficient [41].

In another study, it was shown that the presence of p97, recruited to DNA double-strand breaks by RNF8-generated K48 chains, is a prerequisite for efficient recruitment of downstream DNA damage response proteins BRCA1 (breast cancer type 1 susceptibility protein), 53BP1 and Rad51 [43]. This process is dependent on the NPL4 cofactor [43]. Disruption of p97 by the inhibitor NMS873, or RNA interference of p97, NPL4 was shown in both studies mentioned above to cause foci of DNA damage to persist, indicating that p97 in concert with UFD1–NPL4 is essential for efficient DNA damage repair [41,43].

Analogous to the extraction of Ku from DNA, p97 is also required for the removal of CMG replicative helicases (comprised of Cdc45, MCM2-7 and GINS), those stalled by interstrand cross-links as well as under physiological conditions [44,45]. The stalled replication fork is resolved via a ubiquitin-dependent process where p97 is essential [46]. Again, removal of a protein complex by p97 from chromatin is a prerequisite for access to the DNA lesion and appropriate repair [46]. As a response to a stalled fork, the MCM7 subunit of the helicase is decorated with K48-linked ubiquitin chains, which recruits p97 to extract the helicase. The E3 ubiquitin ligase CRL2^Lrr1^ generates K48-ubiquitinated MCM7, which promotes p97 binding via its UFD1–NPL4 cofactor [47].

In *Caenorhabditis elegans*, an RNAi screen of UBX-containing proteins identified essential p97 cofactors [48]. Both UFD1 and NPL4 are essential for development. In addition, the silencing of FAF1 homolog UBXN3 (UBX-containing protein 3) compromises *C. elegans* survival rates, particularly in p97-depleted cells and those treated with the DNA-damaging agent hydroxyurea [48]. More specifically, UBXN3 binds CDT-1, a DNA replication licensing factor. While CDT-1 is required for replication initiation, it needs to be extracted from chromatin for replication completion. In the absence of p97, or the FAF1 or UFD1–NPL4 cofactors, CDT-1 remains bound to chromatin and severe replication defects are observed [48,49].

In addition to the examples mentioned above, p97 has also been shown to be central to numerous chromatin-related processes beyond the scope of this review, such as extraction of SUMOylated proteins from chromatin and Cockayne syndrome protein extraction to resolve stalled RNA polymerase [50,51], all comprehensively reviewed by ref. [36]. From the studies introduced above, it is apparent that p97 plays a role in the extraction of DNA-binding proteins from different types of DNA damage. The active removal of proteins from chromatin to facilitate access to sites of DNA damage for downstream repair factors, or to allow helicase and polymerase activity to proceed, is a central function of p97. The ATPase is therefore an essential factor in genome stability, reviewed by ref. [52].

### NF-κB activation

The transcription factor NF-κB controls the expression of cytokines, immunoreceptors and other components in the immune system (Figure 1B) [53]. Stimulation of Toll-like receptors or interleukin-1 receptors on the cell surface triggers a cell signaling event utilizing both protein phosphorylation and K63-linked ubiquitination, which leads to the release of NF-κB from the cytosol into the nucleus, where it can affect transcription [54].

In its basal state, the NF-κB heterodimer, consisting of proteins p50 and p65, is kept in an inactive state via association with the inhibitory protein IκBα (NF-κB inhibitor alpha) or related proteins [55]. For the transcription factor to be active, IκBα needs to be degraded, a process which is dependent on p97 [56]. As part of the signaling cascade, both p65 and IκBα become phosphorylated. Subsequent to phosphorylation, which is regulated by an unknown mechanism, the cullin-RING ubiquitin ligase (CRL) CRL1^β-TrCP^ ubiquitinates IκBα and thus recruits p97 [57]. It has been shown that both a functional E3 ubiquitin ligase and active p97 are required for efficient degradation of IκBα and subsequently activation of NF-κB, indicating that p97 is essential for the degradation of ubiquitinated IκBα [57]. There is so far no evidence as to which p97 cofactors, if any, are essential in this pathway. However, the cofactors p47 and FAF1 have inhibitory effects on NF-κB activation [58,59].

### Membrane fusion

The ATPase p97 also plays a role in membrane fusion of most parts of the endomembrane system (Figure 1B). It has functions in the biogenesis of the ER, the Golgi, nuclear membrane assembly and in the fusion of lysosomes. The first cellular functions assigned to p97 were the membrane fusion events essential to Golgi and ER formation [60,61]. The cofactor required for formation of the Golgi, which undergoes disassembly and re-assembly during the cell cyle, was subsequently identified to be p47 [62]. This cofactor contains an N-terminal UBA (ubiquitin-associated) domain, which allows it to bind ubiquitin as well as a C-terminal UBX domain, which allows it to bind p97 [16]. Ubiquitination drives Golgi membrane dynamics [63]. The enzymes driving these ubiquitination events are the E3 ubiquitin ligase HACE1 (HECT domain and ankyrin repeat-containing E3 ubiquitin protein ligase 1) and the DUB VCIP135 (VCP-interacting protein 135 kDa), which act on the t-SNARE (soluble *N*-ethylmaleimide-sensitive fusion protein (NSF) attachment protein receptor) Syn5 (syntaxin5) [64–66]. During early mitosis HACE1 ubiquitinates Syn5, which prevents the interaction of Syn5 with its corresponding v-SNARE Bet1 [66]. A multiprotein complex containing p97 and p47 binds the ubiquitinated SNARE via the UBA domain of p47. In late mitosis, the DUB VCIP135, which associates with p97 via its UBX-L (UBX-like) domain, deubiquitinates Syn5, which permits interaction between the SNAREs, membrane fusion and finally the formation of Golgi cisternae [66].

VCIP135 activity is regulated by protein phosphorylation [67,68]. Phosphomimetic mutants at known VCIP135 phosphorylation sites prevent the association of VCIP135 with p97 [68]. More significantly, VCIP135 phosphorylated at Ser130 displays severely reduced DUB activity [65]. During early mitosis, phosphorylated VCIP135 is inactive and may not be able to bind p97. During late mitosis, VCIP135 becomes dephosphorylated and associates with the p97–p47–Syn5 complex. The active DUB then deubiquitinates Syn5, releasing the SNARE allowing membrane fusion.

This model for membrane fusion differs from the p97 functions described above, as p97 only functions as a scaffold, the role of its ATPase activity has not been fully investigated. There is some circumstantial evidence to support this theory. p47 inhibits p97 ATPase activity by up to 5-fold [69,70]. However, it has also been shown that ATP is required for the p97–p47–VCIP135 complex to dissociate from Syn5 [71].

The p97 cofactor p37, closely related to p47, has also been shown to play a role in Golgi formation. This cofactor contains the C-terminal UBX domain, but has no ubiquitin binding UBA domain [8]. The cofactor is also required for Golgi formation, but a siRNA knockdown of p37 does not affect ERAD [72]. The precise role of p37 in membrane fusion is not as well established as that of p47, but it has been shown that the process requires the tethering protein p115 and its binding partner GM130 [72].

p97 not only has a role in Golgi formation, but is also involved in the assembly of other parts of the endomembrane system. p97 plays a role in the assembly of the nuclear envelope and the ER membrane. The p97 cofactor UFD1 binds CHMP2A (charged multivesicular body protein 2a), a protein that is part of the membrane abscission ESCRT-3 (endosomal sorting complexes required for transport 3) complex. A knockdown of UFD1 prevents correct localization of CHMP2A to the nuclear envelope, suggesting that p97 plays a role in nuclear envelope formation [73]. Analogous to Golgi formation, p97 and its cofactors p47 and VCIP135 are essential to ER formation following mitosis [71].

The p97 cofactors p37, p47 and the DUB VCIP135 play essential roles in membrane fusion. Here, p97 appears to act as a scaffold primarily. While p97 ATPase activity may well have a role in membrane fusion, it has not been described. This is unlike the roles described earlier, where the mechanical energy generated from ATP hydrolysis was used for the extraction of proteins from chromatin, lipid bilayers and protein complexes. Conversely, the ATPase NSF, which is closely related to p97, has been shown to play a central role in membrane fusion. Its ATPase activity is used to disassemble SNAP (soluble NSF attachment protein)–SNARE complexes [74]. It remains to be determined which role p97 ATPase activity has in membrane fusion. Interestingly, it has been shown that p47 inhibits p97, while p37 activates p97 [70].

### Additional roles of p97

There are several additional functions of p97 which do not fall into any of the above categories. In addition to ERAD, p97 also plays a role in the degradation of proteins from the mitochondrial outer membrane. Silencing of the cdc48 gene leads to a slowdown of protein turnover in the mitochondrial membrane and accumulation of ubiquitinated proteins [75]. Analogous to ERAD, the Cdc48–UFD1–NPL4 complex plays a role in mitochondrial degradation [75]. The cofactor Doa1 (degradation of alpha 1), a homolog of human PLAA (phospholipase A-2-activating protein), is essential in cells under mitochondrial oxidative stress, but not under ER stress [75]. This suggests that Doa1 is a cofactor specific to mitochondria-associated degradation.

In another degradation-associated function, p97 is essential for the degradation of aberrant, nascent proteins from ribosomes. mRNAs lacking stop codons lead to stalled ribosomes as the translated poly-A tail produces poly-Lys, which interacts with the ribosomes. The E3 ubiquitin ligase ltn1 (listerin 1) has been identified to ubiquitinate the nascent peptide on stalled ribosome [76,77]. A complex containing p97, UFD1 and NPL4 extracts the peptide from the ribosomes for proteasomal degradation [78].

Just like with many other cellular processes, viral proteins have evolved to take advantage of and hijack p97 function. The influenza A matrix protein M2 undergoes retrotranslocation from the ER lumen to the cytosol [79]. This transport mechanism of M2, which does not rely on M2 ubiquitination, is reliant on the ATPase activity of p97 [79]. Hepatitis E virus protein ORF2 is another viral protein that relies on p97 to be transported from the ER into the cytosol [80]. In both cases, the protein is not readily degraded by the proteasome and remains in the cytosol for several hours.

The protein p97 also has a function in lysosomal trafficking, illustrated by the example of Cav-1 (caveolin-1), a protein which introduces invaginations in the plasma membrane called caveolae [81]. Overexpression of GFP-tagged Cav-1 causes its aggregation in aggresomes in the perinuclear region, which are cleared by a combination of lysosomal and proteasomal degradation. Inhibition of p97, which is found in large amounts in the aggresomes, prevents these structures from being cleared [81]. The lysosomal degradation pathway is also required for the clearance of endogenous Cav-1, which localizes correctly to caveloae [82]. This lysosomal pathway is dependent on p97 and a class of cofactors called Ankrd13 proteins, most of which contain ubiquitin-interacting motifs [82]. The Ankrd13 (ankyrin repeat domain-containing protein 13) UIMs (ubiquitin-interacting motifs) have been shown to bind preferentially to K63-linked ubiquitin chains [82].

## p97 and its cofactors

### Modular architecture of p97 cofactors

**Figure 2.**
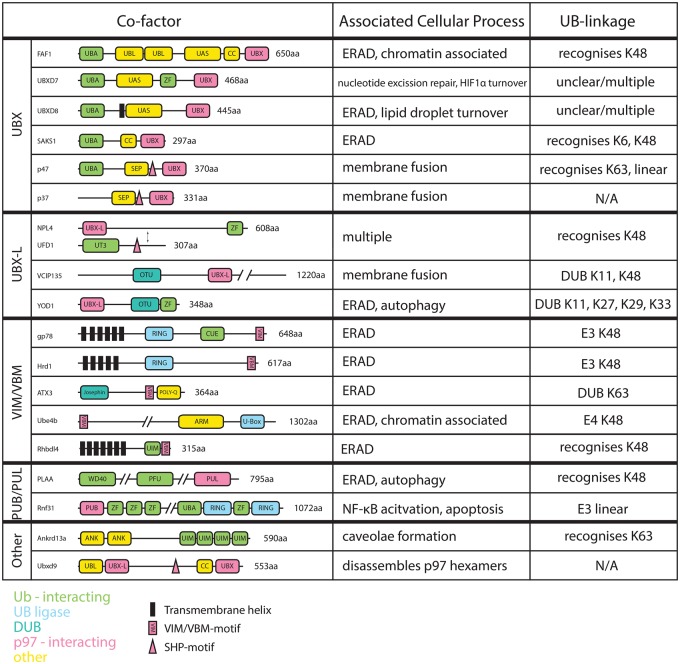
Table of ubiquitin-associated p97 cofactors. Left: domain organization of cofactors, color-coded according to domain function, organized according to p97-interacting motif. Middle: cellular processes the cofactors have been implicated in. Right: types of polyubiquitin the cofactors have been associated with or possesses activity against.

The ATPase p97 is central to a diverse array of cellular processes despite only exhibiting one enzymatic function, the generation of mechanical energy from ATP hydrolysis. The p97 cofactors and their different domain architectures and cellular localizations permit p97 to fulfill different roles depending on the context (Figure 2).

Furthermore, different p97 cofactors have specificity for different ubiquitin linkages. In polyubiquitin, the carboxyl-terminus of a distal ubiquitin is ligated to an amine on the proximal ubiquitin. Ubiquitin contains eight primary amines, seven lysine side chains plus its N-terminus, all of which can be used to form polyubiquitin. Depending on which amine is used, different ubiquitin chains are produced with discrete physical properties and cellular functions, reviewed in ref. [83].

The best characterized polyubiquitin types include K48-, K63- and M1-linked chains. Polyubiquitin linked via K48, the first linkage identified, is a powerful degradation signal [84]. Conversely, K63-linked chains do not constitute a degradation signal, but have a role in signal transduction [85]. Linear polyubiquitin, where a regular peptide bond is formed to link 2 ubiquitin molecules together, plays a role in NF-κB activation and apoptosis [86]. In addition, mixed chains can be formed which may be branched, and ubiquitin itself can be the target of post-translational modifications such as phosphorylation and acetylation [87–90], reviewed in ref. [91].

The cofactors can be divided into two groups depending on what part of p97 they interact with. The larger group of cofactors binds the p97 N-domain, via UBX (ubiquitin regulatory X), UBX-L, VIM, VBM or SHP (binding segment 1) motif, reviewed in ref. [8,9]. A smaller group binds the very C-terminus of p97, via PUB (PNGase/UBA- or UBX-containing proteins) and PUL (PLAA, Ufd3p and Lub1p) domains. As the list of proteins containing p97 recognizing motifs is rather extensive, this review will focus on cofactors also containing ubiquitin-interacting domains.

### UBA–UBX proteins

Of the ∼30 UBX-containing proteins encoded in the human genome, five display a common architecture of an N-terminal UBA domain, which binds ubiquitin, and a C-terminal UBX domain [7]. The proteins UBXD7, UBXD8, FAF1, SAKS1 (SAPK substrate protein 1) and p47 show this domain arrangement. Using immunoprecipitation experiments with all five proteins, the UBX–UBA proteins were shown to be somewhat promiscuous, binding to K11-, K33-, K48- and K63-linked ubiquitin chains to varying degrees, with K11 and K48 chains the most prevalent [92].

The cofactor p47, essential for membrane fusion in Golgi biogenesis, binds p97 via a canonical UBX–N-domain interaction [66,93]. In addition to the UBX domain, p47 also contains an SHP motif. The SHP motif is a more recently discovered p97-interacting sequence, which provides an additional interacting surface for p97 cofactor interactions [17,18]. The UBA domain of p47 has been shown using pull-down experiments to preferentially bind K63 and M1 ubiquitin chains over K48 chains [59].

The adaptor SAKS1 plays a role in ERAD, where it has an inhibitory effect on the clearance of some ERAD substrates, for example α-TCR (T-cell receptor alpha) and NHK-HA (Null Hong Kong variant 1-antitrypsin) [94,95]. The protein recognizes both K48 ubiquitin chains and atypical K6 linked ubiquitin, which can be found on auto-ubiquitinated BRCA1 [94,96].

The UBXD7 cofactor has been implicated in the degradation of cockayne syndrome group protein B, a protein essential in nucleotide excision repair [97]. It also has a role in the turnover of HIF1α (hypoxia-inducible factor 1α) [98]. UBXD7 is required for the interaction of p97 with its substrate HIF1α. In addition, it also interacts with cullin-2, the cognate ubiquitin E3 ligase of HIF1α.

The function of UBXD8 is in the regulation of intracellular fatty acid storage [99]. The protein can be found both in the ER membrane and in cytosolic lipid droplets, where it has a role in the regulation of ATGL (adipose triglyceride lipase) turnover, an enzyme central to triglyceride hydrolysis [100]. The roles of ubiquitin, as well as the molecular role of p97 in this pathway, are still to be determined. UBXD7 and UBXD8 have both been immunoprecipitated with K11, K48 and K63 ubiquitin peptides, but the lack of biochemical data makes it difficult to make firm predictions about the ubiquitin specificity of their UBA domains [98].

The adaptor FAF1, and its interactions with p97 and ubiquitin, have been characterized more comprehensively. A crystal structure of the UBX–N-domain complex shows a canonical interaction [101]. The protein specifically binds K48-linked di-ubiqituin with low micromolar affinity [102]. Pull-down experiments confirm the interaction of FAF1 with K48 ubiquitin and suggest that FAF1 may also bind K63 chains [103]. The coiled-coil region of FAF1 is required for its trimer formation, which may not readily form in solution, but is thought to be formed in complex with p97 [104]. FAF1 also plays a role in ERAD and chromatin-associated degradation [41,48,105]. A p97–FAF1 complex is also essential in the degradation of TGFβ (transforming growth factor beta). Disruption of the FAF1–p97 interaction due to FAF1 phosphorylation by AKT is thought to drive cancer metastasis [106].

### UBX-like domain-containing cofactors

The UFD1–NPL4 complex is an example of a UBX-L domain-containing cofactor. The protein Npl4 contains a UBX-L domain, which binds to the same groove on the p97 N-domain as the UBX domain, but possesses a slightly different fold and employs a distinct binding mode [107]. In addition to the UBX-L of NPL4, UFD1 contains an SHP motif that further stabilizes the interaction with p97 [17,18]. The UFD1–NPL4 heterodimer has been implicated in a multitude of p97-associated processes, primarily ERAD, but also chromatin-associated degradation and ribosome-associated degradation [19,41,78]. Both UFD1 and NPL4 possess ubiquitin-interacting motifs — the NPL4 zinc finger and the UFD1 UT3 domain both contribute to ubiquitin binding. The heterodimer binds to K48-linked ubiquitin chains, but discriminates strongly against K63-linked chains [19].

The DUB VCIP135, central to the regulation of membrane fusion, also binds p97 via a UBX-L domain [71]. It is specifically active against K11- and K48-linked polyubiquitin [108]. In the regulation of membrane fusion however, its substrate is not thought to be polyubiquitin, but the monoubiquitinated t-SNARE Syn5 [66].

Another UBX-L-containing DUB, YOD1, has been associated with lysosomal autophagy and ERAD [33,109]. This DUB is specific against atypical K27, K29 and K33 chains [108].

### VIM/VBM-containing proteins

Unlike the globular UBX and UBX-L folds, the VIM and VBMs are small peptide-like interaction motifs consisting of a single α-helix. The ubiquitin E3 ligases gp78 and Hrd1 both possess such a helical motif — a VIM in the case of gp78 and VBM for Hrd1, reviewed in ref. [9]. Tethered to the ER membrane via their multiple membrane domains, these enzymes play central roles in ERAD [21,25]. In agreement with its role in protein degradation, Hrd1 has E3 ubiquitin ligase activity specific for K48 chains [22]. The E3 ligase gp78 acts on ERAD substrates downstream from Hrd1, presumably after retrotranslocation [27]. This enzyme also catalyzes the formation of K48 chains [19]. The role of this ligase is thought to be the elongation of K48 chains on ERAD substrates. gp78 specifically elongates K48 chains, but does not introduce K48-linked polyubiquitin to other chain types producing mixed chains [27], with its CUE domain acting as a proof-reader in this mechanism [27].

The ubiquitin elongation factor Ube4b is another p97-associated ubiquitin ligase. Its conserved *S. cerevisiae* homolog Ufd2 co-localizes with p97 and proteasomes at sites of DNA damage and has been shown to be essential for the timely removal of Rad51 from such sites [110]. The enzyme also plays a role in ERAD [111]. Ube4b interacts with p97 via its N-terminal VBM [24]. While there is little information about the substrate specificity of Ube4b, there is extensive information about the close homolog Ufd2. The Ufd2 enzyme has the ability to produce various chain types, but most efficiently produces K48 polyubiquitin [112–116]. Interestingly, it strongly interacts with K29 polyubiquitin and adds K48 chains to proximal ubiquitins in such chains to produce branched chains, giving it a ubiquitin-editing function when taking into account the numerous p97-interacting DUBs [116].

The DUB Ataxin-3, which contains a C-terminal VBM, plays a role in ERAD, but its specific function is less clear [30,32]. Its catalytic activity has been reported to negatively regulate ERAD [31], but also be essential for the avoidance of ER stress [32]. The DUB efficiently cleaves K63-linked chains and discriminates against K48 chains; it is particularly effective against longer chains and mixed chains [117]. Ubiquitination of Ataxin-3 itself activates the enzyme, as does binding to p97 [118,119]. Aggregation of Ataxin-3, caused by a polyglutamine sequence C-terminal to the VBM, is known to cause a wide range of neurodegenerative diseases [120].

The protein Rhbdl4 (rhomboid-related protein 4) is a rhomboid protease essential for the ERAD of integral membrane proteins [121]. The protease is embedded in the ER membrane via its seven transmembrane helices and has been shown to cleave ERAD substrates which contain transmembrane helices, in a process that is essential for their efficient degradation [121]. Like other rhomboid proteases, Rhbdl4 is an intramembrane protease, its active site buried in the lipid bilayer where one of its function is the release of growth factors [122]. The protease has been shown to efficiently form a ternary complex with p97 and K48-linked polyubiquitin [123,124]. A VBM, whose interaction with p97 has been comprehensively characterized biochemically, is located at its C-terminus, next to a ubiquitin-interacting motif [123,124].

### PUB/PUL

A small subset of cofactors binds a conserved peptide at the C-terminal end of p97. There are two identified cofactors which bind this peptide and are also associated with the ubiquitin system. The protein PLAA (phospholipase A-2-activating protein) binds p97 via its C-terminal PUL domain and interacts with ubiquitin via its WD40 (WD-repeat) and PFU (PLAA family ubiquitin binding) domains [125–127]. The PLAA cofactor acts in concert with YOD1, Ubxd1 and p97 in the autophagy of damaged lysosomes [109]. PLAA was shown to be required for the clearance of K48-labeled lysosomes, suggesting that it is this linkage type which PLAA recognizes. However, YOD1 depletion also caused K48 lysosome accumulation, an enzyme, which has negligible activity against K48 chains *in vitro* [108,109].

The protein Rnf31 (RING finger protein 31), called HOIP (HOIL-1-interacting protein) in humans, is part of the LUBAC (linear ubiquitin chain assembly complex), the only human UB E3 ligase which catalyzes the formation of linear ubiquitin chains [86,128]. These linear ubiquitin chains are associated with various cellular processes, primarily NF-κB activation and cell death [129]. Rnf31 contains a PUB domain, which recognizes the p97 C-terminal peptide [130]. In addition to p97, the DUB OTULIN also possesses a peptide which interacts with the PUB-binding pocket in the same manner [130]. The physiological significance of these interactions remains to be determined.

### Other significant cofactors

While not *bona fide* ubiquitin-binding p97 cofactors, there are two interesting p97-associated proteins worth introducing. The correct endolysosomal trafficking of the protein caveolin-1 relies on p97 [131]. A ternary complex formed by K63-ubiquitinated Cav-1, p97 and the putative p97 cofactor Ankrd13a is required for correct trafficking [82]. The Ankrd13a protein contains four ubiquitin-interacting motifs which bind K63-linked polyubiquitin [82]. The interaction of Ankrd13a with p97 on the other hand is thought to be indirect, and a cofactor connecting Ankrd13 and p97 has not yet been identified.

Structural biology studies of full-length p97 have identified the enzyme to form stable hexameric rings [3,5]. The p97 cofactor Ubxd9, also called ASPL (alveolar soft part sarcoma locus) or TUG (tether-containing UBX domain for GLUT4), which is located at the ER exit site, has been found to disassemble p97 hexamers, and its overexpression affects an accumulation of ubiquitinated species, consistent with p97 depletion, suggesting that Ubxd9 is a p97 antagonist [132]. Mechanistically, an extended UBX domain is essential for disassembly, the canonical UBX–N-domain interaction forms the initial contact, while α-helical lariats target D1 interprotomer interfaces and cause disassembly [92].

## Structural details of cofactor interactions

A wealth of structural information is available describing p97 and p97-cofactor interactions. While the interactions of all the p97-binding domains have been visualized by X-ray crystallography, the ubiquitin-interacting domains of p97 cofactors unfortunately have not. The mechanisms by which the cofactors discriminate against some ubiquitin chain types still remain to be identified. The following section will therefore focus on the interactions of the p97 N-domain and its binding partners.

### UBX

**Figure 3.**
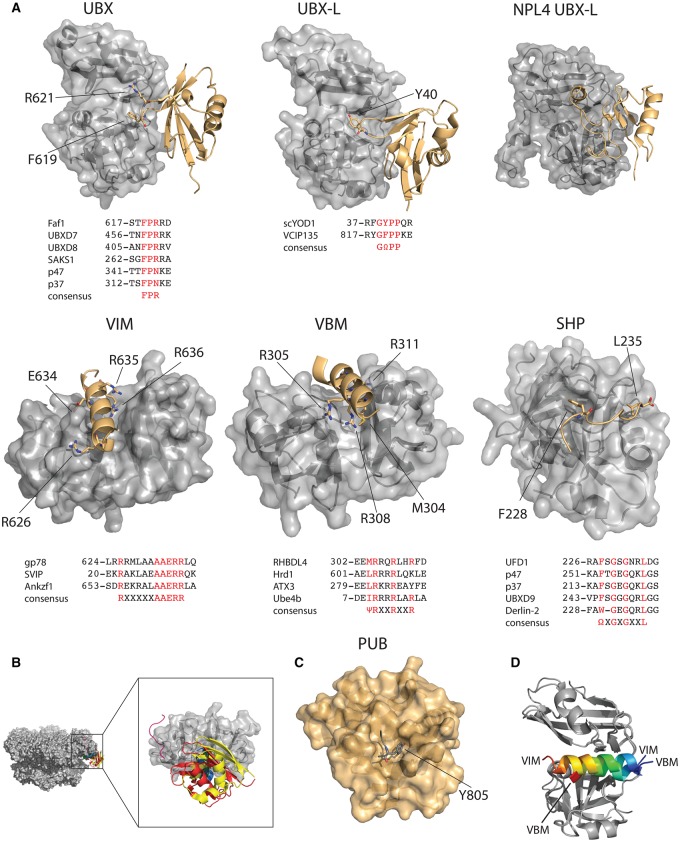
Structural details of p97-cofactor interactions. (**A**) Top: crystal structures of p97 N-domains (gray) in complex with cofactor (gold). FAF1 UBX (PDB: 3QQ8), OTU1 UBX-L (PDB: 4KDL), Npl4 UBX-L (PDB: 2PJH), gp78 VIM (PDB: 3TIW), Rhbdl4 VBM (PDB: 5EPP) and UFD1 SHP (PDB: 5C1B). Conserved residues are indicated. Below: sequence alignment of the conserved interacting motifs, first line in the alignment shows the sequence of the protein shown in the structure above. (**B**) Composite model of UBX (red), UBX-L (yellow), VIM (green), VBM (blue) and SHP (pink) overlayed on the cryo-EM structure of ADP-bound ATP (5FTK). (**C**) Crystal structure of PNGase PUB domain (gold) in complex with p97 C-terminal peptide (gray) (PDB: 2HPJ). (**D**) Overlay of the VIM and VBM complex structures, with the VIM and VBMs rainbow colored from N-terminus (blue) to C-terminus (red). N-domains in gray.

The UBX domain, displaying a ubiquitin-like β-grasp fold with a β-β-α-β-β-α-β topology, binds the p97 N-domain via a conserved loop (Figure 3A). Crystal structures of the complex between the p97 N-domain and the FAF1 and Ubxd7 UBX domain, and between the p97 ND1 and the p47 UBX domain, explain the conserved binding mechanism [93,101,105,133,134]. The binding of the extended UBX domain of Ubxd9 with p97 also includes a canonical UBX–N-domain interaction [92]. The loop connecting β3 and β4 contains an FPR motif, which fits into the groove and is conserved in all UBX domains. The side chain of the conserved phenylalanine residue fits into a hydrophobic pocket located in the groove. The arginine makes contact with an acidic patch on the C-terminal lobe of the N-domain. The central proline takes the *cis*-configuration to enable a rare touch turn to be formed at the end of the loop. The resulting *cis*-peptide has unusual Ψ and Φ angles that enable the Phe and Arg side chains to interact with their respective binding pockets within the N-domain. Using the p47 and FAF1 UBX domains as examples, the affinity of this interaction has been shown to be ∼1 µM [104,135].

### UBX-L

The UBX-L domain also folds into a β-grasp, but with β-β-α-β-β-α-α-β, revealed by the solution structure of rat VCIP135 (PDB:2MX2) and the crystal structure of the *S. cerevisiae* homolog of YOD1 called OTU1 in complex with human p97 (Figure 3A) [136]. The most significant differences between UBX and UBX-L is the insertion of a third helix and the conformation of the β3/β4-turn. Instead of the FPR motif, the UBX-L of OTU1 contains a YPP motif. The tyrosine fits into the same hydrophobic pocket as the phenylalanine of the UBX domain. Again, the central proline is in the *cis*-configuration. The second proline is in the *trans*-configuration. Taken together, the *cis*-proline and its preceding tyrosine residue stabilize the conformation of the touch-turn by π-stacking interactions with each other [136]. The *K*_D_ of the OTU1–p97 interaction was shown by ITC to be 700 nM, indicating that UBX–p97 and UBXL–p97 interactions are similar in strength [136]. It also confirms that this interaction is highly conserved between humans and yeast. In the VCIP UBX-L domain, the tyrosine is replaced by phenylalanine, suggesting a conserved motif of GΩPP, where Ω stands for an aromatic residue [137].

The UBX-L domain of Npl4 shows a similar overall fold, but does not contain a touch-turn motif (Figure 3A) [107]. The β-grasp fold shows a β-β-α-β-α-β topology, and instead of the β3/β4-turn, the interaction with p97 is facilitated by the β-sheet and its adjacent loops. While the binding is considerably weaker than the other OTU1–p97 interaction with a *K*_D_ of 18 µM [105], the overall orientations of the two binding partners are comparable to the other UBX–N-domain interactions.

### VIM

The VIM motif consists of a single α-helix, rich in arginine residues. The crystal structure of the gp78 VIM domain in complex with p97 has revealed the mode of the interaction (Figure 3A) [138]. The helix binds in the groove between the two N-domain lobes, in an interaction that is similar in strength to the UBX interaction. Using the VIM domain of SVIP as an example, the *K*_D_ of this VIM with p97 was in the low micromolar range, similar to UBX and UBX-L binding [138]. The consensus sequence for VIM is RX_5_AAERR, where all three conserved arginines contribute to the binding, the most C-terminal arginine residue contributes to the interaction the most. While the two alanine residues and the glutamate are highly conserved, they are not required for binding, replacement of E634 in the gp78 VIM with a leucine in fact increases the affinity for p97 [138].

### VBM

The VBM, also a helical motif rich in arginine residues, is related to the VIM. A crystal structure of the VBM of RHBDL4 with the p97 N-domain has revealed the interaction of VBM to be highly similar to the VIM–p97 interaction (Figure 3A) [123,124]. The VBM also binds to the groove between the two N-domain lobes, in the same direction as the VIM (Figure 3D). In addition to three conserved arginine residues important for binding, there are also hydrophobic resides that make contact with the same hydrophobic pocket as the conserved phenylalanine of the UBX domain. The consensus motif of the VBM is ΨRXXRXXR, where Ψ stands for an aliphatic residue.

### SHP

The SHP motif is a short linear motif that interacts with the p97 N-domain, but uniquely and not in the aforementioned groove. The consensus motif of SHP is ΩXGXGXXL, where Ω stands for an aromatic residue (Figure 3A) [9,17,18,139,140]. The crystal structures of both the UFD1 and Derlin-1 SHP motifs in complex with the p97 N-domain reveal the interaction to be driven primarily by the two aromatic residues and the conserved leucine, which fit into distinct hydrophobic pockets on the C-terminal lobe of the N-domain [17,18,123,124,140,141]. The second glycine residue and its following phenylalanine make main chain hydrogen bonds with a β-sheet of the N-domain, forming an additional antiparallel β-strand. The binding constant of the SHP–p97 interaction has not been determined, but mutation of essential SHP residues decreases the dissociation constant of the UFD1–NPL4 interaction by a factor of 6- to 17-fold [17,18]. Given that no cofactors have been identified that rely solely on the SHP motif for binding, it is likely that the role of SHP is that of an auxiliary interaction motif only.

From the interactions described, all cofactors bind in the same cleft between the two N-domain lobes. It is likely that *in vivo* there would be competition for the six N-domain-binding sites on each p97 hexamer which could lead to multiple cofactors binding to a single p97 hexamer. While all cofactors described above have been shown to bind to p97 independently, there is evidence that FAF1 and UBXD7 bind preferentially to p97 in complex with UFD1/NPL4 [101]. This suggests that there may be core complexes formed by p97, for examples with UFD1/NPL4 or p47, which are prerequisite to the binding of some ‘secondary’ cofactors [142]. However, it is also possible that temporal and/or spatial differences in the expression and localization of the various cofactors could dictate which p97 complexes are formed [8]. In support of this, p97 can form different complexes *in vitro* with FAF1 and UFD1/NPL4 depending on the concentrations of both cofactors [104].

All of UBX, UBX-L, VIM and VBM bind in the same groove on the N-domain, suggesting that there is competition between cofactors for p97 N-domains, and also placing the cofactors spatially to the ‘side’ of the p97hexamer, towards the N–D1 interface in the ADP-bound form of p97 (Figure 3B). In the ATP-bound form, the cofactors would be located ‘on top’ of the hexamer.

### PUB/PUL

The PUB domain binds a conserved peptide at the extreme C-terminus of p97. The leucine and tyrosine residues that form part of a DDLYG-COOH sequence conserved in eukaryotes bind a hydrophobic pocket on the PUB domain [143]. Protein phosphorylation at Y805 totally abolishes binding of p97 to the PUB domain of PNGase [peptide-*N*(4)-(*N*-acetyl-β-glucosaminyl) asparagine amidase] and also disrupts ERAD (Figure 3C) [143,144]. The observation that Y805 fits tightly into the hydrophobic pocket, with no space for an additional phosphate, provides a clear mechanism as to how this interaction is regulated by phosphorylation. The PUL domain of PLAA, which is structurally unrelated to the PUB domain, binds the C-terminal p97 peptide in a highly similar interaction [127]. While the interaction of the Rnf31 PUB domain with the p97 C-terminal peptide is only ∼50 μM, the DUB OTULIN binds this PUB domain with a dissociation constant ∼300 nM, indicating that this mode of binding can facilitate a wide range of specificities [130].

### Regulation of p97 enzymatic activity by cofactors and *vice versa*

A subset of cofactors do not just bind to p97, but also alter its enzymatic activity. This regulation is effective in both ways, for example the DUB Ataxin-3 has been shown to be activated by p97, while keeping its specificity for K63 chains [119]. This stimulatory effect is absent from disease-associated Ataxin-3 with an extended polyglutamine sequence. The lack of DUB activation and potentially inefficient processing of ERAD substrates may be one mechanism by which the expanded poly-Q of Ataxin-3 may contribute to disease phenotypes [119].

The p37 cofactor activates p97, while p47 decreases ATPase activity [69,70]. Both cofactors result in a tighter *K*_M_ of p97 for its substrate ATP [70]. Interestingly, deletion of an N-terminal region in p47 that lacks homology to p37 turns p47 from an inhibitory cofactor into an activator. These data indicate that UBX domains in general have a stimulatory effect on p97, but an N-terminal region of p47 can counter this effect [70]. Some mutations in the interface between N-domain and D1 domain of p97 are associated with the multisystem disease IBMPFD, defects in ERAD as well as other p97-associated functions, and on a molecular level with altered (increased) ATPase activities [7,145–147].

## Molecular mechanism and enzymology of p97

### Structure

**Figure 4.**
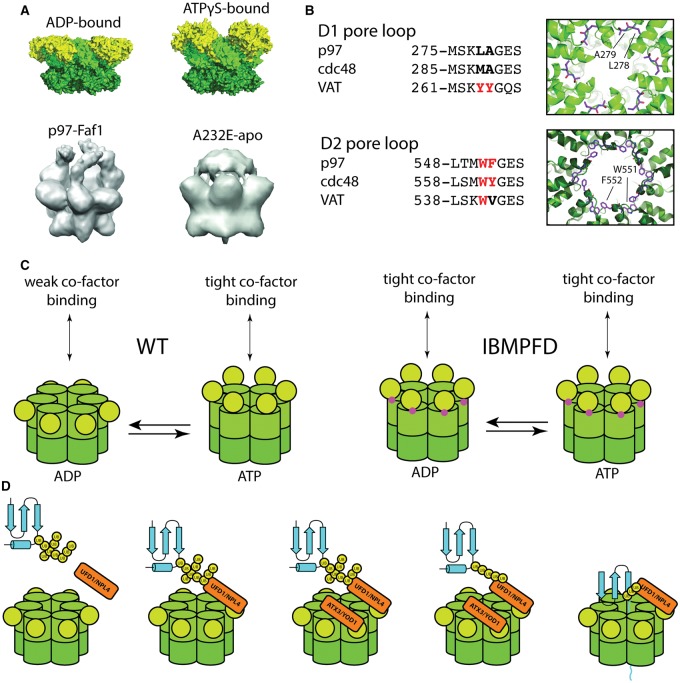
Enzymology of p97. (**A**) EM structures of p97. ADP bound (PDB: 5FTK), ATPγS (PDB: 5FTN), in complex with FAF1 (EMDB-2319) and the IBMPFD mutant A232E (EMDB-2038). (**B**) Left: conservation of D1 and D2 pore loop residues in p97, Cdc48 and VAT. Right: structure of pore loops with residues highlighted on table on the left shown as sticks (PDB: 5FTN). (**C**) Models of p97 conformations in different nucleotide states. p97 domains color-coded as before. IBMPFD mutant positions highlighted in purple. (**D**) Model for p97 activity. p97 forms a complex with UFD1–NPL4 and a polyubiquitinated substrate. Additional cofactors containing DUB activity remove polyubiquitin leaving K48-chains only. The substrate is then threaded through the central pore of p97 and unfolded.

Initially identified as a homohexameric particle by negative stain electron microscopy, p97 was shown to possess ATPase activity that is dependent on its oligomeric state and the presence of Mg^2+^ [5,6]. Subsequently, the crystal structure of the N-domain and first ATPase domain (ND1) was solved by crystallography, followed by full-length p97 [2,3,148]. The hexamer displays a mushroom-like shape where two rings of ATPase domains stack on top of each other and the N-domain is co-planar to the D1 ATPase domain in ADP-bound p97 and in an ‘up’ conformation in the ATP-bound state (Figure 4A) [149]. The D1 and D2 ATPase domains both fold into typical AAA domains with an α/β-subdomain followed by a helical subdomain. Of the 12 ATPase domains in a p97 hexamer, the D1 domains are primarily required for oligomerization, while the D2 domains play a larger role in ATP hydrolysis [150,151]. There is some evidence that the D1–D2 linker, which can introduce asymmetry in the protein, is required for activity — p97 ND1 displays negligible ATPase activity, but a slightly longer protein containing the 20 aa D1–D2 linker possesses ATPase activity roughly half of full-length p97 [146,147]. The six D2 domains of p97 have been shown to bind less than six molecules of ATP, providing an additional link between asymmetry and ATPase activity [152]. ATP hydrolysis is also regulated by inter-protomer interactions between the D2 domain and the C-terminal tail of the neighboring protomer [17,18].

### Molecular function of p97

Some indications as to the molecular function of p97 are provided by its physiological functions and the enzymatic activity of its archaeal homolog VAT. The *T. acidophilum* VAT ATPase, from a prokaryote without a ubiquitin system, has unfoldase activity [153]. Tagging of a protein with an ssrA peptide, a C-terminal degradation tag added to protein lacking an in-frame stop-codon, is essential and sufficient for unfolding by VAT [154]. In VAT, removal of its N-domain increases unfolding activity, indicating that the N-domain may have an autoinhibitory role [153]. Electron microscopy structures of VAT in different nucleotide states suggest that in an ATP-bound VAT, the two ATPase rings form planar rings [155]. However, upon ATP hydrolysis, the 12 ATPase domains can form a continuous spiral. The large structural rearrangement between planar rings and screw-like conformation was proposed to provide the mechanical energy required for protein unfolding [155].

Native human p97 does not possess activity against ssrA-tagged proteins [156]. However, through mutagenesis of VAT, it has been shown that aromatic residues are required in both the D1 and D2 pore for unfolding activity against ssrA-tagged proteins. p97 lacks aromatic residues in the D1 pore, and only through the introduction of such residues and removal of the N-domain could VAT-like unfolding activity be reconstituted (Figure 4B) [156]. Recently, two studies have independently succeeded in reconstituting p97 and Cdc48 unfoldase activity *in vitro*. Both studies used K48-ubiquitinated GFP as a p97 substrate. These were engineered *in vitro*, by enzymatically K48-ubiquitinating either a degron sequence [157] or a ubiquitin [158] linearly fused to the GFP. UFD1–NPL4 was required for efficient unfolding, and YOD1 for substrate release, confirming that p97 relies on this cofactor to recognize K48-ubiquitinated substrates, but removal of ubiquitin is required for complete processing and release [157,158].

The ATPase activity of Cdc48 is reduced to 50% by the presence of Ufd1–NPL4, but increases to 300% by the additional presence of a K48-ubiquitinated substrate, a regulatory mechanism effective at preserving the ATP consumption of such an abundant ATPase [157]. Conversely, the presence of UFD1–NPL4 does not affect human p97 activity [158]. An assay using the engineered substrate also confirmed Cdc48 as a *bona fide* unfoldase whose substrates are threaded through the central pore, as in its prokaryotic homologs (Figure 4D) [157,159]. Given that important pore residues are conserved between Cdc48 and p97, this strongly suggests a highly similar mechanism for mammalian p97 (Figure 4B). These substrates may also aid structural studies and biochemical work to determine the mechanism that provides the mechanical energy for unfolding activity.

### ATPase cycle

Several studies have linked the control of the ATPase cycle to the movement of the N-domain, a regulatory mechanism that appears to fail in IBMPFD mutants, where the ‘up’ conformation is favored even in the apo-form. Structural data on p97 have shown the N-domains to be co-planar with the D1 domain in ADP-bound p97, but in an ‘up’ position in the ATP-bound form [149]. When in complex with the UBX-containing cofactor FAF1, the N-domain also occupies the ‘up’ position (Figure 4A) [104]. The apo-form of the IBMPFD mutant A232E also takes this conformation (Figure 4A) [160]. Biochemical data also support these structural observations. When the N-domain is locked in a co-planar orientation, the ATPase activity is compromised [160]. The cofactors p37 and p47 bind more tightly to the ATP-bound form of p97 than the ADP-bound form. In IBMPFD mutants, the affinity of these cofactors for p97 is independent of nucleotide state, and the interaction is always as tight as for the wild ATP-bound form [161]. Binding of UBX-containing cofactor p37 also increases ATP activity, while p47 inhibits the enzyme [70]. IBMPFD mutants also show higher activity, both in terms of ATP hydrolysis and unfolding activity [158,160]. Interestingly, neither p37 nor p47 is capable of increasing the ATPase activity of IBMPFD mutants of p97 further, providing additional evidence that the N-domain is somehow ‘uncoupled’ from ATP hydrolysis in these disease-linked mutants [70].

Cofactors bind more tightly to hexameric p97 than monomeric N-domain constructs [101]. Given that the N-domain UBX interaction is located on the top of the hexamer, the ‘up’ conformation may favor cofactor binding by virtue of enabling oligomerization of cofactors on ‘top’ of the p97 hexamer. This model has been suggested for the FAF1–p97 and p47–p97 complexes [104].

There also seems to be a correlation between bound ATP, bound cofactor, increased ATP hydrolysis and N-domains in the ‘up’ position, and conversely no ATP bound, weakly binding cofactors and co-planar N-domains (Figure 4C). While there is a strong correlation, it is less clear as to what is the cause and what is the effect. The ATPase activity is also further regulated by extensive post-translational modifications, reviewed by ref. [9]. However, it is clear that the p97 ATP hydrolysis cycle is tightly co-ordinated with cofactor binding and substrate engagement which result in significant conformational changes between the N-domain and D1 and the D1 and D2 AAA domains. It will be important in future studies to try and capture intermediate states of p97 throughout the ATP hydrolysis cycle.

## Therapeutic potential of p97 inhibitors

### Proteotoxic stress in tumour cells

One characteristic of many tumor cell lines is their proteotoxic stress caused by an abundance of misfolded proteins [162]. Owing to their rapid growth and excessive genetic abnormalities, cancer cells suffer from this stress naturally. Enabling an accumulation of misfolded proteins and escalating the proteotoxic stress is an established method to target cancer cells in chemotherapy, proven effective by the proteasome inhibitor bortezomib [162]. However, cancer cells can overcome this selective pressure and develop resistance, for example by up-regulating expression of proteasome subunits as well as chaperones to relieve some of the proteotoxic stress, reviewed in ref. [163]. One molecular mechanism through which this is achieved is via the transcription factor Nrf1 (nuclear respiratory factor 1). The absence of proteasome activity leads to an accumulation of this transcription factor, which triggers increased transcription of proteasome subunits [164]. Targeting other components of the ubiquitin system, such as E3 ubiquitin ligases, DUBs or the interaction surfaces of ubiquitin-binding proteins, has proved challenging, as these proteins rarely possess defined catalytic pockets [165].

Since the ATPase activity of p97 is essential for its function and p97 possesses clearly defined catalytic centers for ATP hydrolysis, inhibition of p97 offers a promising avenue through which proteotoxic stress can be induced. The dependence of tumor cells on p97 activity is further illustrated by the overexpression of this enzyme in NSCLC (non-small-cell lung cancer) cells [166]. Both ATP-competitive and allosteric inhibitors of p97 have been developed with the competitive inhibitors being at a much later stage of development.

### A drug-like ATP-competitive inhibitor of p97

**Figure 5.**
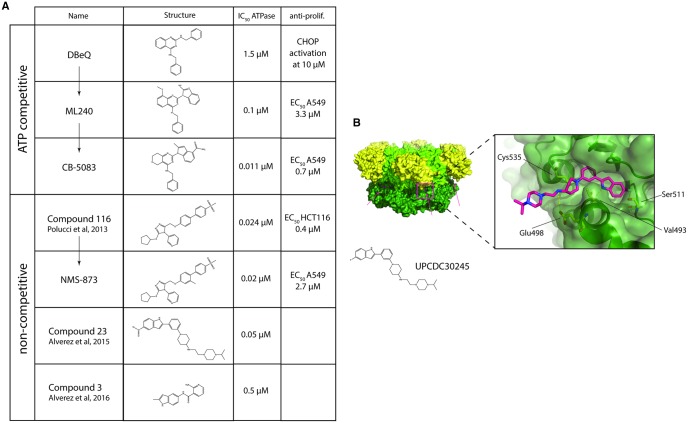
p97 inhibitors. (**A**) Table of a selection of known p97 inhibitors, including name, chemical structure, IC_50_ against p97 ATPase activity and ability to cause an antiproliferative effect in a cell-based assay, e.g. CHOP activation, EC_50_ of cell death against cancer cell lines A549 and HCT116. (**B**) Cryo-EM structure of p97 in complex with the UPCDC30245 inhibitor (pink) (PDB: 5FTJ).

From a high-throughput screen, DBeQ (N2,N4-dibenzylquinazoline-2,4-diamine) was identified as an ATP-competitive p97 inhibitor *in vitro* (Figure 5A). It also successfully inhibited known p97-dependent processes such as ERAD and autophagy [167]. The molecule was developed further using SAR (structure–activity relationship) to produce the more potent inhibitors ML240 and ML241 and finally CB-5083, a potent p97 inhibitor with drug-like properties (Figure 5A) [168–170]. In addition to the inhibition of known p97-dependent cellular processes, CB-5083 was shown to effectively stop tumor growth in both solid and hematological cancer xenografts and has entered Phase I trials in the clinic [170]. Interestingly, CB-5083 reduces the unfolding activity of IBMPFD mutant p97 to that of wild-type p97 [158]. Dendrimer-coated DBeQ has been shown to be more effective in triggering an accumulation of ubiquitinated species than DBeQ by itself, suggesting that coating of a p97 inhibitor, with polymers, may increase effectiveness [171].

### Discovery of noncompetitive inhibitors of p97

Several different classes of allosteric p97 inhibitors have been discovered by high-throughput screening. One of the classes discovered were alkylsulfanyl-1,2,4-triazoles [172]. Following SAR, the most promising lead compound (numbered 116) possessed an ATPase IC_50_ of 24 nM and displayed antiproliferative properties with an IC_50_ value of 380 nM (Figure 5A). The SAR experiments also showed that the IC_50_ values of the ATPase activity and of the antiproliferative effect were not always closely related. Some compounds screened showed strong ATPase inhibition but little antiproliferative effect. The optimized compound showed strong specificity for p97 when tested against other ATPases such as NSF or kinases, but suffered from high clearance when used in an animal model [172].

The inhibitor NMS873 is among the best characterized allosteric p97 inhibitors and was derived from the triazole inhibitors described above (Figure 5A). It has an ATPase IC_50_ comparable to CB5083 [173]. In addition to the p97 inhibitory effect, NMS873 also triggers known phenotypes of p97 inhibition, such as accumulation of polyubiquitin and CHOP induction [173]. While NMS873 is an effective p97 inhibitor *in vitro*, it is at an earlier stage of development than CB5083 in terms of drug-like properties.

In another screen, trifluoromethyl and pentafluorosulfanyl indoles were identified as allosteric p97 inhibitors [174]. Following SAR, the most powerful compound from this class (compound 23) inhibited p97 ATPase activity with a nanomolar IC_50_ (Figure 5A). The structure of p97 in complex with one compound of this class (UPCDC30245) was solved by cryo-EM, confirming that the indole part of the inhibitor interacts with p97 at the D1, D2 interface (Figure 5B) [149].

A third class of allosteric p97 inhibitors discovered by high-throughput screening were indole amides, which act as uncompetitive inhibitors (Figure 5A) [175]. Following SAR, the strongest of the optimized compounds (compound 3) inhibited p97 with an IC_50_ value of up to 500 nM at an ATP concentration of 0.1 mM. In terms of solubility and stability, these indole amides are among the most promising allosteric inhibitors being developed. They do, however, require higher concentrations of compound to effectively inhibit p97. The indole amide failed to display robust antiproliferative effects, but a more potent inhibitor may induce the desired cellular responses [175]. An ATPase IC_50_ considerable lower than 500 nM has been shown to be required for eliciting an antiproliferative effect [172].

In addition to the molecules identified by high-throughput screening, the natural products withaferin A and dehydrocurvularin have been shown to inhibit the enzyme [176,177].

### ATP competitive versus allosteric inhibitors of p97

There are some caveats with ATP competitive inhibition of p97. It has been shown that binding of some p97 cofactors, for example p37 and p47, results in a decreased *K*_M_ for ATP and thus may decrease the potency of a competitive inhibitor [70]. Subsequently, it was reported that for all ATP-competitive inhibitors tested, the IC_50_ of p97–47 is at least 4-fold higher than that of p97 alone. Conversely, the allosteric inhibitor NMS-873 does not lose potency in the presence of p47 [178]. The loss of inhibitor efficacy has so far only been observed for the p97–p47 complex. Subsequent research on the highly similar p97–p37 complex, as well as the ternary p97–UFD1–NPL4 complex, showed that these complexes are equally sensitive to ATP competitive p97 inhibitors as p97 is itself. Furthermore, the discovery that different p97 inhibitors target different complexes with varying efficacies may be the first step towards developing complex-specific inhibitors [178].

### Perspectives on p97 drug discovery efforts

There is a wealth of information on a large number of inhibitors and their interaction with p97 from both *in vivo* and *in vitro* experiments, with the notable absence of any crystal structures of p97-inhibitor complexes which may aid medicinal chemistry efforts. The sole interaction between p97 and an inhibitor has been visualized by high-resolution cryo-electron microscopy [149]. The recent development of *in vitro* substrates for p97 may aid the future development of novel p97 inhibitors [158].

### Trends and conclusions

The diversity of cellular processes listed above illustrates the versatility of p97. The enzyme plays a role in protein degradation from the ER and mitochondrial membrane, primarily in concert with its cofactors UFD1 and NPL4. In addition, it is used to extract ubiquitinated protein complexes from chromatin in DNA repair pathways, DNA replication and transcription. A pathway where its ATPase activity is less well understood is in membrane fusion, where p97 regulates the formation of the endomembrane system. From these well-described processes, there is a common theme among them, which is the recruitment of p97 by ubiquitinated substrates. The vast majority of p97 substrates described are ubiquitinated and their interaction with p97 is mediated by bi-functional cofactors, which contain both p97-interacting and ubiquitin-interacting domains.

Several common themes also become apparent when summarizing the cellular and molecular functions of p97 cofactors. With the exception of the common UBA–UBX architecture, the ubiquitin-associated domains and the p97-interacting motifs occur in many different combinations, highlighting the modularity of these protein domains and binding motifs. While there are a multitude of p97-interacting motifs, most bind the same cleft in the p97 N-domain suggesting competition between cofactors for N-domains. As there are six potential binding sites, there is the possibility of mixed cofactor binding as well as co-operative binding and hierarchical complex assembly. It will be interesting to determine whether multiple complexes can function in similar biochemical pathways or if there is functional specificity assigned to specific sub-complexes.

The different p97-interacting motifs can be found at different positions within p97 cofactors. The UBX and VIM/VBMs are primarily found at the extreme N- and C-termini of the respective cofactor, while the SHP motif is located more centrally in the linear polypeptide chain. Looking at the 3D structures of p97 cofactor complexes reveals that the majority of N-domain-interacting motifs, UBX, UBX-L, VIM and VBM, bind the same groove between the N-domain lobes. Different cofactors thus compete for p97 binding. Hydrophobic and basic residues dominate the consensus sequences of the N-domain-interacting motifs.

In terms of ubiquitin specificity, K48 chains appear the most intimately associated with p97 functions. The ubiquitin-binding cofactors UFD1/NPL4, FAF1, SAKS1, UBXD7 and UBXD8 are all capable of binding K48 polyubiquitin. Only p47, which is not associated with protein degradation or segregation of any kind, but essential to the regulation of membrane fusion, bucks the trend and shows specificity for K63 and linear chains. The ubiquitin specificities of the ubiquitin E3 ligases and DUBs fit into the same pattern as the adaptors. The three ubiquitin ligases Hrd1, gp78 and Ube4b all catalyze the formation of K48-linked chains. The DUBs are somewhat more promiscuous, but neither YOD1, nor ATX3, cleaves K48 isopeptide bonds efficiently. Only VCIP, associated with membrane fusion like p47, is active against K48 polyubiquitin. The cofactor complexes formed by p97 favor the catalysis of K48-linked chains and the hydrolysis of any other type of polyubiquitin. These complexes appear to have evolved to strip p97 substrates of polyubiquitin signals other than K48, from upstream signaling or other processes, and generate or amplify a K48 signal.

Following recruitment of p97 to its substrate, the substrate is unfolded and it is proposed that the unfolded substrate is threaded through the central pore. However, the energy requirements of unfolding larger proteins for threading through the p97 pore are still an open question. It is equally plausible that partial threading results in destablization of target proteins or protein complexes and disassembly or extraction. For a continuous threading mechanism, multiple rounds of ATP hydrolysis would be required and p97 would need to be anchored in such a way as to apply the necessary force for unfolding or extraction. Although *in vitro* using model substrates there is some evidence for threading in Cdc48, it is still unclear how cofactors and ATP hydrolysis orchestrate the disassembly, unfolding process.

In terms of targeting p97 as a drug target for cancer, various screening efforts have produced promising leads with CB5083 being the most developed. Some allosteric inhibitors, for example NMS873, inhibit p97 with comparable potency and elicit antiproliferative responses in cells but are not drug-like, yet. The availability of an *in vitro* substrate may now allow screening for additional inhibitors, which do not interfere with ATPase hydrolysis, but nonetheless disrupt the enzymatic function of p97.

In summary, p97 continues to be a fascinating enzyme and while much has been learned about its structure function and mechanism, there are still a large number of unanswered questions which need to be addressed given the potential clinical importance of p97 as a drug target for cancer treatments.

## Abbreviations

AAA+: ATPases associated with diverse cellular activities
Ankrd13: ankyrin repeat domain-containing protein 13
Ataxin-3: ataxia type 3 protein
BRCA1: breast cancer type 1 susceptibility protein
Cav-1: caveolin-1
Cdc48: cell division cycle protein 48
CDT-1: CDC10-dependent transcript 1
CHMP2A: charged multivesicular body protein 2a
CHOP: C/EBP-homologous protein
CRL: cullin-RING ubiquitin ligase
CUE: coupling of ubiquitin conjugation to ER degradation
DBeQ: N2,N4-dibenzylquinazoline-2,4-diamine
Doa1: degradation of alpha 1
DUBs: deubiquitinases
ERAD: endoplasmic reticulum-associated degradation
FAF1: FAS-associated factor 1
HACE1: HECT domain and ankyrin repeat-containing E3 ubiquitin-protein ligase 1
HIF1α: hypoxia-inducible factor 1α
Hrd1: Hmg2-regulated degradation
IBMPFD: inclusion body myopathy associated with Paget's disease of bone and frontotemporal dementia
IκBα: NF-κB Inhibitor alpha
NF-κB activation: nuclear factor kappa-light-chain-enhancer of activated B cells
NSF: *N*-ethylmaleimide-sensitive fusion protein
OTU1: ovarian tumour domain containing protein 1
PLAA: phospholipase A-2-activating protein
PUB: PNGase/UBA- or UBX-containing proteins
PUL: PLAA, Ufd3p and Lub1p
Rhbdl4: rhomboid-related protein 4
Rnf31: RING finger protein 31
RNF8: RING finger protein 8
SAKS1: SAPK substrate protein 1
SAR: structure–activity relationship
SHP: suppressor of high-copy PP1 protein
SVIP: small VCP-interacting protein
Syn5: syntaxin5
t-SNARE: soluble NSF attachment protein receptor
UBA: ubiquitin-associated
UBX-L: UBX-like
UBXN3: UBX-containing protein 3
UFD1–NPL4: ubiquitin fusion degradation protein 1 and nuclear protein localization protein 4 homolog
UPS: ubiquitin proteasome system
VAT: VCP-like ATPase
VBM: VCP-binding motif
VCIP135: VCP-interacting protein 135 kDa
VCP: valosin-containing protein
VIM: VCP-interacting motif
WD40: WD-repeat
YOD1: yeast OTU domain containing protein

## References

[1] Erzberger J.P. and Berger J.M. (2006) Evolutionary relationships and structural mechanisms of AAA+ proteins. Annu. Rev. Biophys. Biomol. Struct. 35, 93–114 doi:10.1146/annurev.biophys.35.040405.101933 16689629

[2] DeLaBarre B. and Brunger A.T. (2003) Complete structure of p97/valosin-containing protein reveals communication between nucleotide domains. Nat. Struct. Biol. 10, 856–863 doi:10.1038/nsb972 12949490

[3] Huyton T., Pye V.E., Briggs L.C., Flynn T.C., Beuron F., Kondo H. et al. (2003) The crystal structure of murine p97/VCP at 3.6 Å. J. Struct. Biol. 144, 337–348 doi:10.1016/j.jsb.2003.10.007 14643202

[4] Barthelme D. and Sauer R.T. (2016) Origin and functional evolution of the Cdc48/p97/VCP AAA+ protein unfolding and remodeling machine. J. Mol. Biol. 428(9 Pt B), 1861–1869 doi:10.1016/j.jmb.2015.11.015 26608813 PMC4860136

[5] Peters J.M., Walsh M.J. and Franke W.W. (1990) An abundant and ubiquitous homo-oligomeric ring-shaped ATPase particle related to the putative vesicle fusion proteins Sec18p and NSF. EMBO J. 9, 1757–1767 PMID:2140770 10.1002/j.1460-2075.1990.tb08300.x PMC551880

[6] Peters J.-M., Harris J.R., Lustig A., Muller S., Engel A., Volker S. et al. (1992) Ubiquitous soluble Mg^2+^-ATPase complex: a structural study. J. Mol. Biol. 223, 557–571 doi:10.1016/0022-2836(92)90670-F 1531366

[7] Meyer H. and Weihl C.C. (2014) The VCP/p97 system at a glance: connecting cellular function to disease pathogenesis. J. Cell Sci. 127(Pt 18), 3877–3883 doi:10.1242/jcs.093831 25146396 PMC4163641

[8] Kloppsteck P., Ewens C.A., Förster A., Zhang X. and Freemont P.S. (2012) Regulation of p97 in the ubiquitin–proteasome system by the UBX protein-family. Biochim. Biophys. Acta, Mol. Cell Res. 1823, 125–129 doi:10.1016/j.bbamcr.2011.09.006 21963883

[9] Hänzelmann P. and Schindelin H. (2017) The interplay of cofactor interactions and post-translational modifications in the regulation of the AAA+ ATPase p97. Front. Mol. Biosci. 4, 21 doi:10.3389/fmolb.2017.00021 28451587 PMC5389986

[10] Watts G.D.J., Wymer J., Kovach M.J., Mehta S.G., Mumm S., Darvish D. et al. (2004) Inclusion body myopathy associated with Paget disease of bone and frontotemporal dementia is caused by mutant valosin-containing protein. Nat. Genet. 36, 377–381 doi:10.1038/ng1332 15034582

[11] Manno A., Noguchi M., Fukushi J., Motohashi Y. and Kakizuka A. (2010) Enhanced ATPase activities as a primary defect of mutant valosin-containing proteins that cause inclusion body myopathy associated with Paget disease of bone and frontotemporal dementia. Genes Cells 15, 911–922 doi:10.1111/j.1365-2443.2010.01428.x 20604808

[12] Preston G.M. and Brodsky J.L. (2017) The evolving role of ubiquitin modification in endoplasmic reticulum-associated degradation. Biochem. J. 474, 445–469 doi:10.1042/BCJ20160582 28159894 PMC5425155

[13] Ye Y., Shibata Y., Kikkert M., van Voorden S., Wiertz E. and Rapoport T.A. (2005) Recruitment of the p97 ATPase and ubiquitin ligases to the site of retrotranslocation at the endoplasmic reticulum membrane. Proc. Natl Acad. Sci. U.S.A. 102, 14132–14138 doi:10.1073/pnas.0505006102 16186510 PMC1242302

[14] Stein A., Ruggiano A., Carvalho P. and Rapoport T.A. (2014) Key steps in ERAD of luminal ER proteins reconstituted with purified components. Cell 158, 1375–1388 doi:10.1016/j.cell.2014.07.050 25215493 PMC4163015

[15] Hetzer M., Meyer H.H., Walther T.C., Bilbao-Cortes D., Warren G. and Mattaj I.W. (2001) Distinct AAA-ATPase p97 complexes function in discrete steps of nuclear assembly. Nat. Cell Biol. 3, 1086–1091 doi:10.1038/ncb1201-1086 11781570

[16] Meyer H.H., Wang Y. and Warren G. (2002) Direct binding of ubiquitin conjugates by the mammalian p97 adaptor complexes, p47 and Ufd1-Npl4. EMBO J. 21, 5645–5652 doi:10.1093/emboj/cdf579 12411482 PMC131076

[17] Hänzelmann P. and Schindelin H. (2016) Characterization of an additional binding surface on the p97 N-terminal domain involved in bipartite cofactor interactions. Structure 24, 140–147 doi:10.1016/j.str.2015.10.027 26712280

[18] Hänzelmann P. and Schindelin H. (2016) Structural basis of ATP hydrolysis and intersubunit signaling in the AAA+ ATPase p97. Structure 24, 127–139 doi:10.1016/j.str.2015.10.026 26712278

[19] Ye Y., Meyer H.H. and Rapoport T.A. (2003) Function of the p97–Ufd1–Npl4 complex in retrotranslocation from the ER to the cytosol: dual recognition of nonubiquitinated polypeptide segments and polyubiquitin chains. J. Cell Biol. 162, 71–84 doi:10.1083/jcb.200302169 12847084 PMC2172719

[20] Neuber O., Jarosch E., Volkwein C., Walter J. and Sommer T. (2005) Ubx2 links the Cdc48 complex to ER-associated protein degradation. Nat. Cell Biol. 7, 993–998 doi:10.1038/ncb1298 16179953

[21] Bays N.W., Gardner R.G., Seelig L.P., Joazeiro C.A. and Hampton R.Y. (2001) Hrd1p/Der3p is a membrane-anchored ubiquitin ligase required for ER-associated degradation. Nat. Cell Biol. 3, 24–29 doi:10.1038/35050524 11146622

[22] Kikkert M., Doolman R., Dai M., Avner R., Hassink G., van Voorden S. et al. (2004) Human HRD1 is an E3 ubiquitin ligase involved in degradation of proteins from the endoplasmic reticulum. J. Biol. Chem. 279, 3525–3534 doi:10.1074/jbc.M307453200 14593114

[23] Gauss R., Sommer T. and Jarosch E. (2006) The Hrd1p ligase complex forms a linchpin between ER-lumenal substrate selection and Cdc48p recruitment. EMBO J. 25, 1827–1835 doi:10.1038/sj.emboj.7601088 16619026 PMC1456945

[24] Morreale G., Conforti L., Coadwell J., Wilbrey A.L. and Coleman M.P. (2009) Evolutionary divergence of valosin-containing protein/cell division cycle protein 48 binding interactions among endoplasmic reticulum-associated degradation proteins. FEBS J. 276, 1208–1220 doi:10.1111/j.1742-4658.2008.06858.x 19175675

[25] Fang S., Ferrone M., Yang C., Jensen J.P., Tiwari S. and Weissman A.M. (2001) The tumor autocrine motility factor receptor, gp78, is a ubiquitin protein ligase implicated in degradation from the endoplasmic reticulum. Proc. Natl Acad. Sci. U.S.A. 98, 14422–14427 doi:10.1073/pnas.251401598 11724934 PMC64697

[26] Ballar P., Shen Y., Yang H. and Fang S. (2006) The role of a novel p97/valosin-containing protein-interacting motif of gp78 in endoplasmic reticulum-associated degradation. J. Biol. Chem. 281, 35359–35368 doi:10.1074/jbc.M603355200 16987818

[27] Zhang T., Xu Y., Liu Y. and Ye Y. (2015) Gp78 functions downstream of Hrd1 to promote degradation of misfolded proteins of the endoplasmic reticulum. Mol. Biol. Cell 26, 4438–4450 doi:10.1091/mbc.E15-06-0354 26424800 PMC4666138

[28] Koch P., Breuer P., Peitz M., Jungverdorben J., Kesavan J., Poppe D. et al. (2011) Excitation-induced ataxin-3 aggregation in neurons from patients with Machado–Joseph disease. Nature 480, 543–546 doi:10.1038/nature10671 22113611

[29] Doss-Pepe E.W., Stenroos E.S., Johnson W.G. and Madura K. (2003) Ataxin-3 interactions with rad23 and valosin-containing protein and its associations with ubiquitin chains and the proteasome are consistent with a role in ubiquitin-mediated proteolysis. Mol. Cell. Biol. 23, 6469–6483 doi:10.1128/MCB.23.18.6469-6483.2003 12944474 PMC193705

[30] Boeddrich A., Gaumer S., Haacke A., Tzvetkov N., Albrecht M., Evert B.O. et al. (2006) An arginine/lysine-rich motif is crucial for VCP/p97-mediated modulation of ataxin-3 fibrillogenesis. EMBO J. 25, 1547–1558 doi:10.1038/sj.emboj.7601043 16525503 PMC1440312

[31] Zhong X. and Pittman R.N. (2006) Ataxin-3 binds VCP/p97 and regulates retrotranslocation of ERAD substrates. Hum. Mol. Genet. 15, 2409–2420 doi:10.1093/hmg/ddl164 16822850

[32] Wang Q., Li L. and Ye Y. (2006) Regulation of retrotranslocation by p97-associated deubiquitinating enzyme ataxin-3. J. Cell Biol. 174, 963–971 doi:10.1083/jcb.200605100 17000876 PMC2064388

[33] Ernst R., Mueller B., Ploegh H.L. and Schlieker C. (2009) The otubain YOD1 is a deubiquitinating enzyme that associates with p97 to facilitate protein dislocation from the ER. Mol. Cell 36, 28–38 doi:10.1016/j.molcel.2009.09.016 19818707 PMC2774717

[34] Liu Y. and Ye Y. (2013) Roles of p97-associated deubiquitinases in protein quality control at the endoplasmic reticulum. Curr. Protein Pept. Sci. 13, 436–446 doi:10.2174/138920312802430608 PMC3483884 22812527

[35] Locke M., Toth J.I. and Petroski M.D. (2014) Lys^11^- and Lys^48^-linked ubiquitin chains interact with p97 during endoplasmic-reticulum-associated degradation. Biochem. J. 459, 205–216 doi:10.1042/BJ20120662 24417208 PMC4160081

[36] Franz A., Ackermann L. and Hoppe T. (2016) Ring of change: CDC48/p97 drives protein dynamics at chromatin. Front. Genet. 7, 73 doi:10.3389/fgene.2016.00073 27200082 PMC4853748

[37] Polo S.E. and Jackson S.P. (2011) Dynamics of DNA damage response proteins at DNA breaks: a focus on protein modifications. Genes Dev. 25, 409–433 doi:10.1101/gad.2021311 21363960 PMC3049283

[38] Schwertman P., Bekker-Jensen S. and Mailand N. (2016) Regulation of DNA double-strand break repair by ubiquitin and ubiquitin-like modifiers. Nat. Rev. Mol. Cell Biol. 17, 379–394 doi:10.1038/nrm.2016.58 27211488

[39] Huen M.S.Y., Grant R., Manke I., Minn K., Yu X., Yaffe M.B. et al. (2007) RNF8 transduces the DNA-damage signal via histone ubiquitylation and checkpoint protein assembly. Cell 131, 901–914 doi:10.1016/j.cell.2007.09.041 18001825 PMC2149842

[40] Taccioli G.E., Gottlieb T.M., Blunt T., Priestley A., Demengeot J., Mizuta R. et al. (1994) Ku80: product of the XRCC5 gene and its role in DNA repair and V(D)J recombination. Science 265, 1442–1445 doi:10.1126/science.8073286 8073286

[41] van den Boom J., Wolf M., Weimann L., Schulze N., Li F., Kaschani F. et al. (2016) VCP/p97 extracts sterically trapped Ku70/80 rings from DNA in double-strand break repair. Mol. Cell 64, 189–198 doi:10.1016/j.molcel.2016.08.037 27716483 PMC5161236

[42] Walker J.R., Corpina R.A. and Goldberg J. (2001) Structure of the Ku heterodimer bound to DNA and its implications for double-strand break repair. Nature 412, 607–614 doi:10.1038/35088000 11493912

[43] Meerang M., Ritz D., Paliwal S., Garajova Z., Bosshard M., Mailand N. et al. (2011) The ubiquitin-selective segregase VCP/p97 orchestrates the response to DNA double-strand breaks. Nat. Cell Biol. 13, 1376–1382 doi:10.1038/ncb2367 22020440

[44] Maric M., Maculins T., De Piccoli G. and Labib K. (2014) Cdc48 and a ubiquitin ligase drive disassembly of the CMG helicase at the end of DNA replication. Science 346, 1253596 doi:10.1126/science.1253596 25342810 PMC4300516

[45] Moreno S.P., Bailey R., Campion N., Herron S. and Gambus A. (2014) Polyubiquitylation drives replisome disassembly at the termination of DNA replication. Science 346, 477–481 doi:10.1126/science.1253585 25342805

[46] Fullbright G., Rycenga H.B., Gruber J.D. and Long D.T. (2016) p97 promotes a conserved mechanism of helicase unloading during DNA cross-link repair. Mol. Cell. Biol. 36, 2983–2994 doi:10.1128/MCB.00434-16 27644328 PMC5108885

[47] Dewar J.M., Low E., Mann M., Räschle M. and Walter J.C. (2017) CRL2^Lrr1^ promotes unloading of the vertebrate replisome from chromatin during replication termination. Genes Dev. 31, 275–290 doi:10.1101/gad.291799.116 28235849 PMC5358724

[48] Franz A., Pirson P.A., Pilger D., Halder S., Achuthankutty D., Kashkar H. et al. (2016) Chromatin-associated degradation is defined by UBXN-3/FAF1 to safeguard DNA replication fork progression. Nat. Commun. 7, 10612 doi:10.1038/ncomms10612 26842564 PMC4743000

[49] Franz A., Orth M., Pirson P.A., Sonneville R., Blow J.J., Gartner A. et al. (2011) CDC-48/p97 coordinates CDT-1 degradation with GINS chromatin dissociation to ensure faithful DNA replication. Mol. Cell 44, 85–96 doi:10.1016/j.molcel.2011.08.028 21981920 PMC3428722

[50] Nie M., Aslanian A., Prudden J., Heideker J., Vashisht A.A., Wohlschlegel J.A. et al. (2012) Dual recruitment of Cdc48 (p97)-Ufd1-Npl4 ubiquitin-selective segregase by small ubiquitin-like modifier protein (SUMO) and ubiquitin in SUMO-targeted ubiquitin ligase-mediated genome stability functions. J. Biol. Chem. 287, 29610–29619 doi:10.1074/jbc.M112.379768 22730331 PMC3436128

[51] Bergink S., Ammon T., Kern M., Schermelleh L., Leonhardt H. and Jentsch S. (2013) Role of Cdc48/p97 as a SUMO-targeted segregase curbing Rad51–Rad52 interaction. Nat. Cell Biol. 15, 526–532 doi:10.1038/ncb2729 23624404

[52] Vaz B., Halder S. and Ramadan K. (2013) Role of p97/VCP (Cdc48) in genome stability. Front. Genet. 4, 60 doi:10.3389/fgene.2013.00060 23641252 PMC3639377

[53] Pahl H.L. (1999) Activators and target genes of Rel/NF-κB transcription factors. Oncogene 18, 6853–6866 doi:10.1038/sj.onc.1203239 10602461

[54] Chen Z.J. (2012) Ubiquitination in signaling to and activation of IKK. Immunol. Rev. 246, 95–106 doi:10.1111/j.1600-065X.2012.01108.x 22435549 PMC3549672

[55] Henkel T., Machleidt T., Alkalay I., Krönke M., Ben-Neriah Y. and Baeuerle P.A. (1993) Rapid proteolysis of IκB-α is necessary for activation of transcription factor NF-κB. Nature 365, 182–185 doi:10.1038/365182a0 8371761

[56] Dai R.-M., Chen E., Longo D.L., Gorbea C.M. and Li C.-C.H. (1998) Involvement of valosin-containing protein, an ATPase co-purified with IκBα and 26 S proteasome, in ubiquitin-proteasome-mediated degradation of IκBα. J. Biol. Chem. 273, 3562–3573 doi:10.1074/jbc.273.6.3562 9452483

[57] Schweitzer K., Pralow A. and Naumann M. (2016) p97/VCP promotes Cullin-RING-ubiquitin-ligase/proteasome-dependent degradation of IκBα and the preceding liberation of RelA from ubiquitinated IκBα. J. Cell Mol. Med. 20, 58–70 doi:10.1111/jcmm.12702 26463447 PMC4717852

[58] Kinoshita T., Kondoh C., Hasegawa M., Imamura R. and Suda T. (2006) Fas-associated factor 1 is a negative regulator of PYRIN-containing Apaf-1-like protein 1. Int. Immunol. 18, 1701–1706 doi:10.1093/intimm/dxl104 17046979

[59] Shibata Y., Oyama M., Kozuka-Hata H., Han X., Tanaka Y., Gohda J.-i. et al. (2012) p47 negatively regulates IKK activation by inducing the lysosomal degradation of polyubiquitinated NEMO. Nat. Commun. 3, 1061 doi:10.1038/ncomms2068 22990857

[60] Latterich M., Fröhlich K.-U. and Schekman R. (1995) Membrane fusion and the cell cycle: Cdc48p participates in the fusion of ER membranes. Cell 82, 885–893 doi:10.1016/0092-8674(95)90268-6 7553849

[61] Rabouille C., Levine T.P., Peters J.-M. and Warren G. (1995) An NSF-like ATPase, p97, and NSF mediate cisternal regrowth from mitotic Golgi fragments. Cell 82, 905–914 doi:10.1016/0092-8674(95)90270-8 7553851

[62] Kondo H., Rabouille C., Newman R., Levine T.P., Pappin D., Freemont P. et al. (1997) p47 is a cofactor for p97-mediated membrane fusion. Nature 388, 75–78 doi:10.1038/40411 9214505

[63] Meyer H.H. (2005) Golgi reassembly after mitosis: the AAA family meets the ubiquitin family. Biochim. Biophys. Acta, Mol. Cell Res. 1744, 108–119 doi:10.1016/j.bbamcr.2005.03.011 15878210

[64] Tang D., Xiang Y., De Renzis S., Rink J., Zheng G., Zerial M. et al. (2011) The ubiquitin ligase HACE1 regulates Golgi membrane dynamics during the cell cycle. Nat. Commun. 2, 501 doi:10.1038/ncomms1509 21988917 PMC3282116

[65] Zhang X. and Wang Y. (2015) Cell cycle regulation of VCIP135 deubiquitinase activity and function in p97/p47-mediated Golgi reassembly. Mol. Biol. Cell 26, 2242–2251 doi:10.1091/mbc.E15-01-0041 25904330 PMC4462942

[66] Huang S., Tang D. and Wang Y. (2016) Monoubiquitination of syntaxin 5 regulates Golgi membrane dynamics during the cell cycle. Dev. Cell 38, 73–85 doi:10.1016/j.devcel.2016.06.001 27404360 PMC4942811

[67] Totsukawa G., Matsuo A., Kubota A., Taguchi Y. and Kondo H. (2013) Mitotic phosphorylation of VCIP135 blocks p97ATPase-mediated Golgi membrane fusion. Biochem. Biophys. Res. Commun. 433, 237–242 doi:10.1016/j.bbrc.2013.02.090 23500464

[68] Zhang X., Zhang H. and Wang Y. (2014) Phosphorylation regulates VCIP135 function in Golgi membrane fusion during the cell cycle. J. Cell Sci. 127(Pt 1), 172–181 doi:10.1242/jcs.134668 24163436 PMC3874786

[69] Meyer H.H., Kondo H. and Warren G. (1998) The p47 cofactor regulates the ATPase activity of the membrane fusion protein, p97. FEBS Lett. 437, 255–257 doi:10.1016/S0014-5793(98)01232-0 9824302

[70] Zhang X., Gui L., Zhang X., Bulfer S.L., Sanghez V., Wong D.E. et al. (2015) Altered cofactor regulation with disease-associated p97/VCP mutations. Proc. Natl Acad. Sci. U.S.A. 112, E1705–E1714 doi:10.1073/pnas.1418820112 25775548 PMC4394316

[71] Uchiyama K., Jokitalo E., Kano F., Murata M., Zhang X., Canas B. et al. (2002) VCIP135, a novel essential factor for p97/p47-mediated membrane fusion, is required for Golgi and ER assembly in vivo. J. Cell Biol. 159, 855–866 doi:10.1083/jcb.200208112 12473691 PMC2173386

[72] Uchiyama K., Totsukawa G., Puhka M., Kaneko Y., Jokitalo E., Dreveny I. et al. (2006) p37 is a p97 adaptor required for Golgi and ER biogenesis in interphase and at the end of mitosis. Dev. Cell 11, 803–816 doi:10.1016/j.devcel.2006.10.016 17141156

[73] Olmos Y., Hodgson L., Mantell J., Verkade P. and Carlton J.G. (2015) ESCRT-III controls nuclear envelope reformation. Nature 522, 236–239 doi:10.1038/nature14503 26040713 PMC4471131

[74] Zhao M., Wu S., Zhou Q., Vivona S., Cipriano D.J., Cheng Y. et al. (2015) Mechanistic insights into the recycling machine of the SNARE complex. Nature 518, 61–67 doi:10.1038/nature14148 25581794 PMC4320033

[75] Wu X., Li L. and Jiang H. (2016) Doa1 targets ubiquitinated substrates for mitochondria-associated degradation. J. Cell Biol. 213, 49–63 doi:10.1083/jcb.201510098 27044889 PMC4828692

[76] Bengtson M.H. and Joazeiro C.A.P. (2010) Role of a ribosome-associated E3 ubiquitin ligase in protein quality control. Nature 467, 470–473 doi:10.1038/nature09371 20835226 PMC2988496

[77] Defenouillère Q., Zhang E., Namane A., Mouaikel J., Jacquier A. and Fromont-Racine M. (2016) Rqc1 and Ltn1 prevent C-terminal alanine-threonine tail (CAT-tail)-induced protein aggregation by efficient recruitment of Cdc48 on stalled 60S subunits. J. Biol. Chem. 291, 12245–12253 doi:10.1074/jbc.M116.722264 27129255 PMC4933273

[78] Verma R., Oania R.S., Kolawa N.J. and Deshaies R.J. (2013) Cdc48/p97 promotes degradation of aberrant nascent polypeptides bound to the ribosome. eLife 2, e00308 doi:10.7554/eLife.00308 23358411 PMC3552423

[79] Bhowmick S., Chakravarty C., Sellathamby S. and Lal S.K. (2017) The influenza A virus matrix protein 2 undergoes retrograde transport from the endoplasmic reticulum into the cytoplasm and bypasses cytoplasmic proteasomal degradation. Arch. Virol. 162, 919–929 doi:10.1007/s00705-016-3153-8 27942972

[80] Surjit M., Jameel S. and Lal S.K. (2007) Cytoplasmic localization of the ORF2 protein of hepatitis E virus is dependent on its ability to undergo retrotranslocation from the endoplasmic reticulum. J. Virol. 81, 3339–3345 doi:10.1128/JVI.02039-06 17229684 PMC1866057

[81] Tiwari A., Copeland C.A., Han B., Hanson C.A., Raghunathan K. and Kenworthy A.K. (2016) Caveolin-1 is an aggresome-inducing protein. Sci. Rep. 6, 38681 doi:10.1038/srep38681 27929047 PMC5144149

[82] Burana D., Yoshihara H., Tanno H., Yamamoto A., Saeki Y., Tanaka K. et al. (2016) The Ankrd13 family of ubiquitin-interacting motif-bearing proteins regulates valosin-containing protein/p97 protein-mediated lysosomal trafficking of caveolin 1. J. Biol. Chem. 291, 6218–6231 doi:10.1074/jbc.M115.710707 26797118 PMC4813590

[83] Akutsu M., Dikic I. and Bremm A. (2016) Ubiquitin chain diversity at a glance. J. Cell Sci. 129, 875–880 doi:10.1242/jcs.183954 26906419

[84] Chau V., Tobias J.W., Bachmair A., Marriott D., Ecker D.J., Gonda D.K. et al. (1989) A multiubiquitin chain is confined to specific lysine in a targeted short-lived protein. Science 243, 1576–1583 doi:10.1126/science.2538923 2538923

[85] Hoege C., Pfander B., Moldovan G.-L., Pyrowolakis G. and Jentsch S. (2002) RAD6-dependent DNA repair is linked to modification of PCNA by ubiquitin and SUMO. Nature 419, 135–141 doi:10.1038/nature00991 12226657

[86] Kirisako T., Kamei K., Murata S., Kato M., Fukumoto H., Kanie M. et al. (2006) A ubiquitin ligase complex assembles linear polyubiquitin chains. EMBO J. 25, 4877–4887 doi:10.1038/sj.emboj.7601360 17006537 PMC1618115

[87] Ben-Saadon R., Zaaroor D., Ziv T. and Ciechanover A. (2006) The polycomb protein Ring1B generates self atypical mixed ubiquitin chains required for its *in vitro* histone H2A ligase activity. Mol. Cell 24, 701–711 doi:10.1016/j.molcel.2006.10.022 17157253

[88] Koyano F., Okatsu K., Kosako H., Tamura Y., Go E., Kimura M. et al. (2014) Ubiquitin is phosphorylated by PINK1 to activate parkin. Nature 510, 162–166 doi:10.1038/nature13392 24784582

[89] Meyer H.-J. and Rape M. (2014) Enhanced protein degradation by branched ubiquitin chains. Cell 157, 910–921 doi:10.1016/j.cell.2014.03.037 24813613 PMC4028144

[90] Ohtake F., Saeki Y., Sakamoto K., Ohtake K., Nishikawa H., Tsuchiya H. et al. (2015) Ubiquitin acetylation inhibits polyubiquitin chain elongation. EMBO Rep. 16, 192–201 doi:10.15252/embr.201439152 25527407 PMC4328746

[91] Ohtake F. and Tsuchiya H. (2017) The emerging complexity of ubiquitin architecture. J. Biochem. 161, 125–133 doi:10.1093/jb/mvw088 28011818

[92] Arumughan A., Roske Y., Barth C., Forero L.L., Bravo-Rodriguez K., Redel A. et al. (2016) Quantitative interaction mapping reveals an extended UBX domain in ASPL that disrupts functional p97 hexamers. Nat. Commun. 7, 13047 doi:10.1038/ncomms13047 27762274 PMC5080433

[93] Dreveny I., Kondo H., Uchiyama K., Shaw A., Zhang X. and Freemont P.S. (2004) Structural basis of the interaction between the AAA ATPase p97/VCP and its adaptor protein p47. EMBO J. 23, 1030–1039 doi:10.1038/sj.emboj.7600139 14988733 PMC380986

[94] LaLonde D.P. and Bretscher A. (2011) The UBX protein SAKS1 negatively regulates endoplasmic reticulum-associated degradation and p97-dependent degradation. J. Biol. Chem. 286, 4892–4901 doi:10.1074/jbc.M110.158030 21135095 PMC3039385

[95] Park E.S., Yoo Y.J. and Elangovan M. (2017) The opposite role of two UBA–UBX containing proteins, p47 and SAKS1 in the degradation of a single ERAD substrate, α-TCR. Mol. Cell. Biochem. 425, 37–45 doi:10.1007/s11010-016-2860-5 27785701

[96] Wu-Baer F., Ludwig T. and Baer R. (2010) The UBXN1 protein associates with autoubiquitinated forms of the BRCA1 tumor suppressor and inhibits its enzymatic function. Mol. Cell. Biol. 30, 2787–2798 doi:10.1128/MCB.01056-09 20351172 PMC2876507

[97] He J., Zhu Q., Wani G., Sharma N. and Wani A.A. (2016) Valosin-containing protein (VCP)/p97 segregase mediates proteolytic processing of Cockayne Syndrome Group B (CSB) in damaged chromatin. J. Biol. Chem. 291, 7396–7408 doi:10.1074/jbc.M115.705350 26826127 PMC4817171

[98] Alexandru G., Graumann J., Smith G.T., Kolawa N.J., Fang R. and Deshaies R.J. (2008) UBXD7 binds multiple ubiquitin ligases and implicates p97 in HIF1α turnover. Cell 134, 804–816 doi:10.1016/j.cell.2008.06.048 18775313 PMC2614663

[99] Lee J.N., Kim H., Yao H., Chen Y., Weng K. and Ye J. (2010) Identification of Ubxd8 protein as a sensor for unsaturated fatty acids and regulator of triglyceride synthesis. Proc. Natl Acad. Sci. U.S.A. 107, 21424–21429 doi:10.1073/pnas.1011859107 21115839 PMC3003070

[100] Olzmann J.A., Richter C.M. and Kopito R.R. (2013) Spatial regulation of UBXD8 and p97/VCP controls ATGL-mediated lipid droplet turnover. Proc. Natl Acad. Sci. U.S.A. 110, 1345–1350 doi:10.1073/pnas.1213738110 23297223 PMC3557085

[101] Hänzelmann P., Buchberger A. and Schindelin H. (2011) Hierarchical binding of cofactors to the AAA ATPase p97. Structure 19, 833–843 doi:10.1016/j.str.2011.03.018 21645854

[102] Song J.S., Park J.K., Lee J.-J., Choi Y.-S., Ryu K.-S., Kim J.-H. et al. (2009) Structure and interaction of ubiquitin-associated domain of human Fas-associated factor 1. Protein Sci. 18, 2265–2276 doi:10.1002/pro.237 19722279 PMC2788281

[103] Song E.J., Yim S.-H., Kim E., Kim N.-S. and Lee K.-J. (2005) Human Fas-associated factor 1, interacting with ubiquitinated proteins and valosin-containing protein, is involved in the ubiquitin-proteasome pathway. Mol. Cell. Biol. 25, 2511–2524 doi:10.1128/MCB.25.6.2511-2524.2005 15743842 PMC1061599

[104] Ewens C.A., Panico S., Kloppsteck P., McKeown C., Ebong I.-O., Robinson C. et al. (2014) The p97-FAF1 protein complex reveals a common mode of p97 adaptor binding. J. Biol. Chem. 289, 12077–12084 doi:10.1074/jbc.M114.559591 24619421 PMC4002113

[105] Lee J.-J., Park J.K., Jeong J., Jeon H., Yoon J.-B., Kim E.E.K. et al. (2013) Complex of Fas-associated factor 1 (FAF1) with valosin-containing protein (VCP)-Npl4-Ufd1 and polyubiquitinated proteins promotes endoplasmic reticulum-associated degradation (ERAD). J. Biol. Chem. 288, 6998–7011 doi:10.1074/jbc.M112.417576 23293021 PMC3591610

[106] Xie F., Jin K., Shao L., Fan Y., Tu Y., Li Y. et al. (2017) FAF1 phosphorylation by AKT accumulates TGF-β type II receptor and drives breast cancer metastasis. Nat. Commun. 8, 15021 doi:10.1038/ncomms15021 28443643 PMC5414047

[107] Isaacson R.L., Pye V.E., Simpson P., Meyer H.H., Zhang X., Freemont P.S. et al. (2007) Detailed structural insights into the p97-Npl4-Ufd1 interface. J. Biol. Chem. 282, 21361–21369 doi:10.1074/jbc.M610069200 17491009

[108] Mevissen T.E.T., Hospenthal M.K., Geurink P.P., Elliott P.R., Akutsu M., Arnaudo N. et al. (2013) OTU deubiquitinases reveal mechanisms of linkage specificity and enable ubiquitin chain restriction analysis. Cell 154, 169–184 doi:10.1016/j.cell.2013.05.046 23827681 PMC3705208

[109] Papadopoulos C., Kirchner P., Bug M., Grum D., Koerver L., Schulze N. et al. (2017) VCP/p97 cooperates with YOD1, UBXD1 and PLAA to drive clearance of ruptured lysosomes by autophagy. EMBO J. 36, 135–150 doi:10.15252/embj.201695148 27753622 PMC5242375

[110] Ackermann L., Schell M., Pokrzywa W., Kevei E., Gartner A., Schumacher B. et al. (2016) E4 ligase–specific ubiquitination hubs coordinate DNA double-strand-break repair and apoptosis. Nat. Struct. Mol. Biol. 23, 995–1002 doi:10.1038/nsmb.3296 27669035 PMC5349472

[111] Sirisaengtaksin N., Gireud M., Yan Q., Kubota Y., Meza D., Waymire J.C. et al. (2014) UBE4B protein couples ubiquitination and sorting machineries to enable epidermal growth factor receptor (EGFR) degradation. J. Biol. Chem. 289, 3026–3039 doi:10.1074/jbc.M113.495671 24344129 PMC3908433

[112] Koegl M., Hoppe T., Schlenker S., Ulrich H.D., Mayer T.U. and Jentsch S. (1999) A novel ubiquitination factor, E4, is involved in multiubiquitin chain assembly. Cell 96, 635–644 doi:10.1016/S0092-8674(00)80574-7 10089879

[113] Hatakeyama S., Yada M., Matsumoto M., Ishida N. and Nakayama K.-I. (2001) U box proteins as a new family of ubiquitin-protein ligases. J. Biol. Chem. 276, 33111–33120 doi:10.1074/jbc.M102755200 11435423

[114] Saeki Y., Tayama Y., Toh-e A. and Yokosawa H. (2004) Definitive evidence for Ufd2-catalyzed elongation of the ubiquitin chain through Lys48 linkage. Biochem. Biophys. Res. Commun. 320, 840–845 doi:10.1016/j.bbrc.2004.05.216 15240124

[115] Richly H., Rape M., Braun S., Rumpf S., Hoege C. and Jentsch S. (2005) A series of ubiquitin binding factors connects CDC48/p97 to substrate multiubiquitylation and proteasomal targeting. Cell 120, 73–84 doi:10.1016/j.cell.2004.11.013 15652483

[116] Liu C., Liu W., Ye Y. and Li W. (2017) Ufd2p synthesizes branched ubiquitin chains to promote the degradation of substrates modified with atypical chains. Nat. Commun. 8, 14274 doi:10.1038/ncomms14274 28165462 PMC5303827

[117] Winborn B.J., Travis S.M., Todi S.V., Scaglione K.M., Xu P., Williams A.J. et al. (2008) The deubiquitinating enzyme ataxin-3, a polyglutamine disease protein, edits Lys^63^ linkages in mixed linkage ubiquitin chains. J. Biol. Chem. 283, 26436–26443 doi:10.1074/jbc.M803692200 18599482 PMC2546540

[118] Todi S.V., Winborn B.J., Scaglione K.M., Blount J.R., Travis S.M. and Paulson H.L. (2009) Ubiquitination directly enhances activity of the deubiquitinating enzyme ataxin-3. EMBO J. 28, 372–382 doi:10.1038/emboj.2008.289 19153604 PMC2646149

[119] Laço M.N., Cortes L., Travis S.M., Paulson H.L. and Rego A.C. (2012) Valosin-containing protein (VCP/p97) is an activator of wild-type ataxin-3. PLoS ONE 7, e43563 doi:10.1371/journal.pone.0043563 22970133 PMC3435318

[120] Li X., Liu H., Fischhaber P.L. and Tang T.-S. (2015) Toward therapeutic targets for SCA3: Insight into the role of Machado–Joseph disease protein ataxin-3 in misfolded proteins clearance. Prog. Neurobiol. 132, 34–58 doi:10.1016/j.pneurobio.2015.06.004 26123252

[121] Fleig L., Bergbold N., Sahasrabudhe P., Geiger B., Kaltak L. and Lemberg M.K. (2012) Ubiquitin-dependent intramembrane rhomboid protease promotes ERAD of membrane proteins. Mol. Cell 47, 558–569 doi:10.1016/j.molcel.2012.06.008 22795130

[122] Wunderle L., Knopf J.D., Kuhnle N., Morle A., Hehn B., Adrain C. et al. (2016) Rhomboid intramembrane protease RHBDL4 triggers ER-export and non-canonical secretion of membrane-anchored TGFα. Sci. Rep. 6, 27342 doi:10.1038/srep27342 27264103 PMC4893610

[123] Lim J.J., Lee Y., Ly T.T., Kang J.Y., Lee J.-G., An J.Y. et al. (2016) Structural insights into the interaction of p97 N-terminus domain and VBM in rhomboid protease, RHBDL4. Biochem. J. 473, 2863–2880 doi:10.1042/BCJ20160237 27407164

[124] Lim J.J., Lee Y., Yoon S.Y., Ly T.T., Kang J.Y., Youn H.-S. et al. (2016) Structural insights into the interaction of human p97 N-terminal domain and SHP motif in Derlin-1 rhomboid pseudoprotease. FEBS Lett. 590, 4402–4413 doi:10.1002/1873-3468.12447 27714797

[125] Mullally J.E., Chernova T. and Wilkinson K.D. (2006) Doa1 is a Cdc48 adapter that possesses a novel ubiquitin binding domain. Mol. Cell. Biol. 26, 822–830 doi:10.1128/MCB.26.3.822-830.2006 16428438 PMC1347030

[126] Pashkova N., Gakhar L., Winistorfer S.C., Yu L., Ramaswamy S. and Piper R.C. (2010) WD40 repeat propellers define a ubiquitin-binding domain that regulates turnover of F box proteins. Mol. Cell 40, 433–443 doi:10.1016/j.molcel.2010.10.018 21070969 PMC3266742

[127] Qiu L., Pashkova N., Walker J.R., Winistorfer S., Allali-Hassani A., Akutsu M. et al. (2010) Structure and function of the PLAA/Ufd3-p97/Cdc48 complex. J. Biol. Chem. 285, 365–372 doi:10.1074/jbc.M109.044685 19887378 PMC2804184

[128] Stieglitz B., Rana R.R., Koliopoulos M.G., Morris-Davies A.C., Schaeffer V., Christodoulou E. et al. (2013) Structural basis for ligase-specific conjugation of linear ubiquitin chains by HOIP. Nature 503, 422–426 doi:10.1038/nature12638 24141947 PMC3838313

[129] Rittinger K. and Ikeda F. (2017) Linear ubiquitin chains: enzymes, mechanisms and biology. Open Biol. 7, 170026 doi:10.1098/rsob.170026 28446710 PMC5413910

[130] Schaeffer V., Akutsu M., Olma M.H., Gomes L.C., Kawasaki M. and Dikic I. (2014) Binding of OTULIN to the PUB domain of HOIP controls NF-κB signaling. Mol. Cell 54, 349–361 doi:10.1016/j.molcel.2014.03.016 24726327

[131] Ritz D., Vuk M., Kirchner P., Bug M., Schütz S., Hayer A. et al. (2011) Endolysosomal sorting of ubiquitylated caveolin-1 is regulated by VCP and UBXD1 and impaired by VCP disease mutations. Nat. Cell Biol. 13, 1116–1123 doi:10.1038/ncb2301 21822278 PMC3246400

[132] Orme C.M. and Bogan J.S. (2012) The ubiquitin regulatory X (UBX) domain-containing protein TUG regulates the p97 ATPase and resides at the endoplasmic reticulum-Golgi intermediate compartment. J. Biol. Chem. 287, 6679–6692 doi:10.1074/jbc.M111.284232 22207755 PMC3307297

[133] Kim K.H., Kang W., Suh S.W. and Yang J.K. (2011) Crystal structure of FAF1 UBX domain in complex with p97/VCP N domain reveals a conformational change in the conserved FcisP touch-turn motif of UBX domain. Proteins Struct. Funct. Bioinf. 79, 2583–2587 doi:10.1002/prot.23073 21739474

[134] Li Z.-H., Wang Y., Xu M. and Jiang T. (2017) Crystal structures of the UBX domain of human UBXD7 and its complex with p97 ATPase. Biochem. Biophys. Res. Commun. 486, 94–100 doi:10.1016/j.bbrc.2017.03.005 28274878

[135] Beuron F., Dreveny I., Yuan X., Pye V.E., McKeown C., Briggs L.C. et al. (2006) Conformational changes in the AAA ATPase p97–p47 adaptor complex. EMBO J. 25, 1967–1976 doi:10.1038/sj.emboj.7601055 16601695 PMC1456939

[136] Kim S.J., Cho J., Song E.J., Kim S.J., Kim H.M., Lee K.E. et al. (2014) Structural basis for ovarian tumor domain-containing protein 1 (OTU1) binding to p97/valosin-containing protein (VCP). J. Biol. Chem. 289, 12264–12274 doi:10.1074/jbc.M113.523936 24610782 PMC4007425

[137] Aasland R., Abrams C., Ampe C., Ball L.J., Bedford M.T., Cesareni G. et al. (2002) Normalization of nomenclature for peptide motifs as ligands of modular protein domains. FEBS Lett. 513, 141–144 doi:10.1016/S0014-5793(01)03295-1 11911894

[138] Hänzelmann P. and Schindelin H. (2011) The structural and functional basis of the p97/valosin-containing protein (VCP)-interacting motif (VIM): mutually exclusive binding of cofactors to the N-terminal domain of p97. J. Biol. Chem. 286, 38679–38690 doi:10.1074/jbc.M111.274506 21914798 PMC3207442

[139] Bruderer R.M., Brasseur C. and Meyer H.H. (2004) The AAA ATPase p97/VCP interacts with its alternative co-factors, Ufd1-Npl4 and p47, through a common bipartite binding mechanism. J. Biol. Chem. 279, 49609–49616 doi:10.1074/jbc.M408695200 15371428

[140] Le L.T.M., Kang W., Kim J.-Y., Le O.T.T., Lee S.Y. and Yang J.K. (2016) Structural details of Ufd1 binding to p97 and their functional implications in ER-associated degradation. PLoS ONE 11, e0163394 doi:10.1371/journal.pone.0163394 27684549 PMC5042407

[141] Greenblatt E.J., Olzmann J.A. and Kopito R.R. (2011) Derlin-1 is a rhomboid pseudoprotease required for the dislocation of mutant α-1 antitrypsin from the endoplasmic reticulum. Nat. Struct. Mol. Biol. 18, 1147–1152 doi:10.1038/nsmb.2111 21909096 PMC3196324

[142] Meyer H. (2012) P97 complexes as signal integration hubs. BMC Biol. 10, 48 doi:10.1186/1741-7007-10-48 22694940 PMC3374291

[143] Zhao G., Zhou X., Wang L., Li G., Schindelin H. and Lennarz W.J. (2007) Studies on peptide:N-glycanase-p97 interaction suggest that p97 phosphorylation modulates endoplasmic reticulum-associated degradation. Proc. Natl Acad. Sci. U.S.A. 104, 8785–8790 doi:10.1073/pnas.0702966104 17496150 PMC1885580

[144] Li G., Zhao G., Schindelin H. and Lennarz W.J. (2008) Tyrosine phosphorylation of ATPase p97 regulates its activity during ERAD. Biochem. Biophys. Res. Commun. 375, 247–251 doi:10.1016/j.bbrc.2008.08.018 18706391

[145] Weihl C.C., Dalal S., Pestronk A. and Hanson P.I. (2006) Inclusion body myopathy-associated mutations in p97/VCP impair endoplasmic reticulum-associated degradation. Hum. Mol. Genet. 15, 189–199 doi:10.1093/hmg/ddi426 16321991

[146] Tang W.K., and Xia D. (2016) Mutations in the human AAA+ chaperone p97 and related diseases. Front. Mol. Biosci. 3, 79 doi:10.3389/fmolb.2016.00079 27990419 PMC5131264

[147] Tang W.K. and Xia D. (2016) Role of the D1-D2 linker of human VCP/p97 in the asymmetry and ATPase activity of the D1-domain. Sci. Rep. 6, 20037 doi:10.1038/srep20037 26818443 PMC4730245

[148] Zhang X., Shaw A., Bates P.A., Newman R.H., Gowen B., Orlova E. et al. (2000) Structure of the AAA ATPase p97. Mol. Cell 6, 1473–1484 doi:10.1016/S1097-2765(00)00143-X 11163219

[149] Banerjee S., Bartesaghi A., Merk A., Rao P., Bulfer S.L., Yan Y. et al. (2016) 2.3 a resolution cryo-EM structure of human p97 and mechanism of allosteric inhibition. Science 351, 871–875 doi:10.1126/science.aad7974 26822609 PMC6946184

[150] Song C., Wang Q. and Li C.-C.H. (2003) ATPase activity of p97-valosin-containing protein (VCP). D2 mediates the major enzyme activity, and D1 contributes to the heat-induced activity. J. Biol. Chem. 278, 3648–3655 doi:10.1074/jbc.M208422200 12446676

[151] Wang Q., Song C. and Li C.-C.H. (2003) Hexamerization of p97-VCP is promoted by ATP binding to the D1 domain and required for ATPase and biological activities. Biochem. Biophys. Res. Commun. 300, 253–260 doi:10.1016/S0006-291X(02)02840-1 12504076

[152] Briggs L.C., Baldwin G.S., Miyata N., Kondo H., Zhang X. and Freemont P.S. (2008) Analysis of nucleotide binding to p97 reveals the properties of a tandem AAA hexameric ATPase. J. Biol. Chem. 283, 13745–13752 doi:10.1074/jbc.M709632200 18332143 PMC2376215

[153] Gerega A., Rockel B., Peters J., Tamura T., Baumeister W. and Zwickl P. (2005) VAT, the thermoplasma homolog of mammalian p97/VCP, is an N domain-regulated protein unfoldase. J. Biol. Chem. 280, 42856–42862 doi:10.1074/jbc.M510592200 16236712

[154] Keiler K.C., Waller P.R.H. and Sauer R.T. (1996) Role of a peptide tagging system in degradation of proteins synthesized from damaged messenger RNA. Science 271, 990–993 doi:10.1126/science.271.5251.990 8584937

[155] Huang R., Ripstein Z.A., Augustyniak R., Lazniewski M., Ginalski K., Kay L.E. et al. (2016) Unfolding the mechanism of the AAA+ unfoldase VAT by a combined cryo-EM, solution NMR study. Proc. Natl Acad. Sci. U.S.A. 113, E4190–E4199 doi:10.1073/pnas.1603980113 27402735 PMC4961139

[156] Rothballer A., Tzvetkov N. and Zwickl P. (2007) Mutations in p97/VCP induce unfolding activity. FEBS Lett. 581, 1197–1201 doi:10.1016/j.febslet.2007.02.031 17346713

[157] Bodnar N.O. and Rapoport T.A. (2017) Molecular mechanism of substrate processing by the Cdc48 ATPase complex. Cell 169, 722–735.e9 doi:10.1016/j.cell.2017.04.020 28475898 PMC5751438

[158] Blythe E.E., Olson K.C., Chau V. and Deshaies R.J. (2017) Ubiquitin- and ATP-dependent unfoldase activity of P97/VCP•NPLOC4•UFD1L is enhanced by a mutation that causes multisystem proteinopathy. Proc. Natl Acad. Sci. U.S.A. 114, E4380–E4388 doi:10.1073/pnas.1706205114 28512218 PMC5465906

[159] Hanson P.I. and Whiteheart S.W. (2005) AAA+ proteins: have engine, will work. Nat. Rev. Mol. Cell Biol. 6, 519–529 doi:10.1038/nrm1684 16072036

[160] Niwa H., Ewens C.A., Tsang C., Yeung H.O., Zhang X. and Freemont P.S. (2012) The role of the N-domain in the ATPase activity of the mammalian AAA ATPase p97/VCP. J. Biol. Chem. 287, 8561–8570 doi:10.1074/jbc.M111.302778 22270372 PMC3318706

[161] Bulfer S.L., Chou T.-F. and Arkin M.R. (2016) P97 disease mutations modulate nucleotide-induced conformation to alter protein–protein interactions. ACS Chem. Biol. 11, 2112–2116 doi:10.1021/acschembio.6b00350 27267671 PMC5224236

[162] Deshaies R.J. (2014) Proteotoxic crisis, the ubiquitin-proteasome system, and cancer therapy. BMC Biol. 12, 94 doi:10.1186/s12915-014-0094-0 25385277 PMC4226866

[163] Lü S. and Wang J. (2013) The resistance mechanisms of proteasome inhibitor bortezomib. Biomark. Res. 1, 13 doi:10.1186/2050-7771-1-13 24252210 PMC4177604

[164] Radhakrishnan S.K., Lee C.S., Young P., Beskow A., Chan J.Y. and Deshaies R.J. (2010) Transcription factor Nrf1 mediates the proteasome recovery pathway after proteasome inhibition in mammalian cells. Mol. Cell 38, 17–28 doi:10.1016/j.molcel.2010.02.029 20385086 PMC2874685

[165] Huang X. and Dixit V.M. (2016) Drugging the undruggables: exploring the ubiquitin system for drug development. Cell Res. 26, 484–498 doi:10.1038/cr.2016.31 27002218 PMC4822129

[166] Valle C.W., Min T., Bodas M., Mazur S., Begum S., Tang D. et al. (2011) Critical role of VCP/p97 in the pathogenesis and progression of non-small cell lung carcinoma. PLoS ONE 6, e29073 doi:10.1371/journal.pone.0029073 22216170 PMC3245239

[167] Chou T.-F., Brown S.J., Minond D., Nordin B.E., Li K., Jones A.C. et al. (2011) Reversible inhibitor of p97, DBeQ, impairs both ubiquitin-dependent and autophagic protein clearance pathways. Proc. Natl Acad. Sci. U.S.A. 108, 4834–4839 doi:10.1073/pnas.1015312108 21383145 PMC3064330

[168] Chou T.-F., Li K., Frankowski K.J., Schoenen F.J. and Deshaies R.J. (2013) Structure-activity relationship study reveals ML240 and ML241 as potent and selective inhibitors of p97 ATPase. Chem. Med. Chem. 8, 297–312 doi:10.1002/cmdc.201200520 23316025 PMC3662613

[169] Anderson D.J., Le Moigne R., Djakovic S., Kumar B., Rice J., Wong S. et al. (2015) Targeting the AAA ATPase p97 as an approach to treat cancer through disruption of protein homeostasis. Cancer Cell 28, 653–665 doi:10.1016/j.ccell.2015.10.002 26555175 PMC4941640

[170] Zhou H.-J., Wang J., Yao B., Wong S., Djakovic S., Kumar B. et al. (2015) Discovery of a first-in-class, potent, selective, and orally bioavailable inhibitor of the p97 AAA ATPase (CB-5083). J. Med. Chem. 58, 9480–9497 doi:10.1021/acs.jmedchem.5b01346 26565666

[171] Walworth K., Bodas M., Campbell R.J., Swanson D., Sharma A. and Vij N. (2016) Dendrimer-based selective proteostasis-inhibition strategy to control NSCLC growth and progression. PLoS ONE 11, e0158507 doi:10.1371/journal.pone.0158507 27434122 PMC4951140

[172] Polucci P., Magnaghi P., Angiolini M., Asa D., Avanzi N., Badari A. et al. (2013) Alkylsulfanyl-1,2,4-triazoles, a new class of allosteric valosine containing protein inhibitors. Synthesis and structure–activity relationships. J. Med. Chem. 56, 437–450 doi:10.1021/jm3013213 23245311

[173] Magnaghi P., D'Alessio R., Valsasina B., Avanzi N., Rizzi S., Asa D. et al. (2013) Covalent and allosteric inhibitors of the ATPase VCP/p97 induce cancer cell death. Nat. Chem. Biol. 9, 548–556 doi:10.1038/nchembio.1313 23892893

[174] Alverez C., Arkin M.R., Bulfer S.L., Colombo R., Kovaliov M., LaPorte M.G. et al. (2015) Structure–activity study of bioisosteric trifluoromethyl and pentafluorosulfanyl indole inhibitors of the AAA ATPase p97. ACS Med. Chem. Lett. 6, 1225–1230 doi:10.1021/acsmedchemlett.5b00364 26713109 PMC4677369

[175] Alverez C., Bulfer S.L., Chakrasali R., Chimenti M.S., Deshaies R.J., Green N. et al. (2016) Allosteric indole amide inhibitors of p97: identification of a novel probe of the ubiquitin pathway. ACS Med. Chem. Lett. 7, 182–187 doi:10.1021/acsmedchemlett.5b00396 26985295 PMC4753542

[176] Tao S., Tillotson J., Wijeratne E.M.K., Xu Y.-m., Kang M.J., Wu T. et al. (2015) Withaferin A analogs that target the AAA+ chaperone p97. ACS Chem. Biol. 10, 1916–1924 doi:10.1021/acschembio.5b00367 26006219 PMC4593394

[177] Tillotson J., Bashyal B.P., Kang M.J., Shi T., De La Cruz F., Gunatilaka A.A.L. et al. (2016) Selective inhibition of p97 by chlorinated analogues of dehydrocurvularin. Org. Biomol. Chem. 14, 5918–5921 doi:10.1039/C6OB00560H 27223265 PMC5466822

[178] Gui L., Zhang X., Li K., Frankowski K.J., Li S., Wong D.E. et al. (2016) Evaluating p97 inhibitor analogues for potency against p97-p37 and p97-Npl4-Ufd1 complexes. Chem. Med. Chem. 11, 953–957 doi:10.1002/cmdc.201600036 27043824 PMC9049307

